# Low‐gluten, nontransgenic wheat engineered with CRISPR/Cas9

**DOI:** 10.1111/pbi.12837

**Published:** 2017-11-24

**Authors:** Susana Sánchez‐León, Javier Gil‐Humanes, Carmen V. Ozuna, María J. Giménez, Carolina Sousa, Daniel F. Voytas, Francisco Barro

**Affiliations:** ^1^ Departamento de Mejora Genética Vegetal Instituto de Agricultura Sostenible (IAS‐CSIC) Córdoba Spain; ^2^ Department of Genetics Cell Biology, and Development Center for Genome Engineering University of Minnesota Minneapolis MN USA; ^3^ Departamento de Microbiología y Parasitología Facultad de Farmacia Universidad de Sevilla Sevilla Spain

**Keywords:** coeliac disease, α‐gliadins, CRISPR/Cas9

## Abstract

Coeliac disease is an autoimmune disorder triggered in genetically predisposed individuals by the ingestion of gluten proteins from wheat, barley and rye. The α‐gliadin gene family of wheat contains four highly stimulatory peptides, of which the 33‐mer is the main immunodominant peptide in patients with coeliac. We designed two sgRNAs to target a conserved region adjacent to the coding sequence for the 33‐mer in the α‐gliadin genes. Twenty‐one mutant lines were generated, all showing strong reduction in α‐gliadins. Up to 35 different genes were mutated in one of the lines of the 45 different genes identified in the wild type, while immunoreactivity was reduced by 85%. Transgene‐free lines were identified, and no off‐target mutations have been detected in any of the potential targets. The low‐gluten, transgene‐free wheat lines described here could be used to produce low‐gluten foodstuff and serve as source material to introgress this trait into elite wheat varieties.

## Introduction

Wheat is one of the most widely grown crops in the world and a major component of the human diet. Wheat grain contains gluten proteins, which are responsible for the unique viscoelastic properties of wheat‐derived foods; however, they also trigger certain pathologies in susceptible individuals. Amongst these, the α‐gliadin family is the main protein group associated with the development of coeliac disease and noncoeliac gluten sensitivity, which affect more than 7% of the Western population (Mustalahti *et al*., 2010; Sapone *et al*., 2011). In bread wheat, α‐gliadins are encoded by approximately 100 genes and pseudogenes (Ozuna *et al*., 2015) organized in tandem at the *Gli‐2* loci of chromosomes 6A, 6B and 6D. Traditional mutagenesis and plant breeding have failed to obtain low immunogenic wheat varieties for patients with coeliac. Here, we show that CRISPR/Cas9 technology can be used to precisely and efficiently reduce the amount of α‐gliadins in the seed kernel, providing bread and durum wheat lines with reduced immunoreactivity for gluten‐intolerant consumers.

## Results and discussion

**Figure 1:**
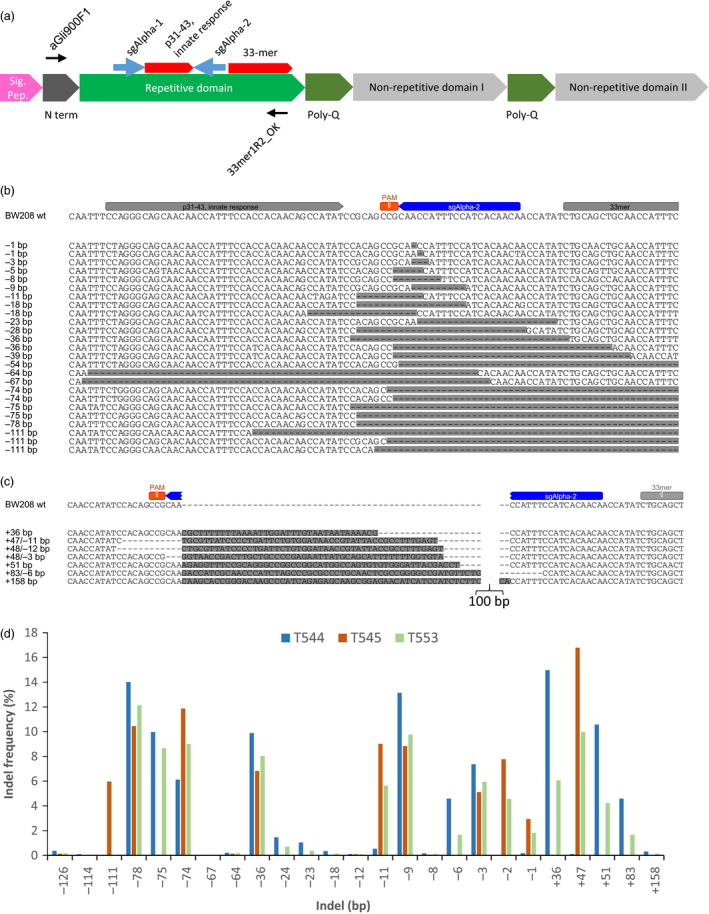
Gene editing of α‐gliadins in bread wheat. (a) Schematic of a typical α‐gliadin gene indicating the different protein domains. Two of the peptide sequences involved in gluten intolerance (p31‐43 and the 33‐mer) are represented by red arrows, whereas the target sequences for the sgRNAs (sgAlpha‐1 and sgAlpha‐2) are represented by blue arrows. Black arrows indicate primers used for Illumina sequencing. (b–d) Illumina sequencing of the α‐gliadin genes of 3 T1 BW208 mutant lines (T544, T545 and T553) transformed with sgAlpha‐2. (b) Alignment of the different deletion types found at the target locus of sgAlpha‐2; (c) Alignment of the different insertions at the target locus of sgAlpha‐2; and (d) frequency of the different type of insertions and deletions.

To precisely modify the immunoreactive α‐gliadin genes, we designed two sgRNAs (sgAlpha‐1 and sgAlpha‐2) (Figure 1a) to target conserved regions adjacent to the coding sequence for the immunodominant epitope in wheat gluten, a protease‐resistant, 33‐amino acid peptide that contains six overlapping copies of three distinct, tandemly organized epitopes (DQ2.5‐glia‐α1a, PFPQPELPY; DQ2.5‐glia‐α2, PQPELPYPQ; and DQ2.5‐glia‐α1b, PYPQPELPY) (Tye‐Din *et al*., 2010). The CRISPR/Cas9 constructs were transformed into two bread wheat (BW028 and TAH53) and one durum wheat (DP) cultivars, resulting in twenty‐one (15 bread wheat and 6 durum wheat) T0 transgenic lines. DNA was isolated from leaves of 17 T1 transgenic plants (5 BW208, 4 TAH53, and 8 DP) and the corresponding wild‐type varieties, and PCR amplicons encompassing the sgAlpha‐1 and sgAlpha‐2 target sites were subjected to Illumina high‐throughput DNA sequencing (Figure 1a, Table S1). We observed considerable variability in the bread wheat and durum wheat wild‐type sequences, due to randomly distributed SNPs and differences in the number of encoded epitopes in the 33‐mer region (Figure S1). As expected, a number of sequences were pseudogenes with premature stop codons, and frameshift mutations in the C‐terminus (Figure S2). We found 45, 52 and 43 different α‐gliadin sequences that were highly represented (frequencies higher than 0.3%) in BW208, THA53 and DP, respectively. Of these, 35, 13 and 29 were, respectively, mutated by CRISPR/Cas9 (Figure S3). The mutation spectrum in the α‐gliadins was characterized in the various T1 transgenic plants (Figure 1b–d, Table S2, Figures S4–S6). Due to the presence of the Cas9 expression vector in some of the mutant lines, the frequency of mutations observed might be overestimated, as a consequence of somatic mutations. However, in most cases we observed similar mutation frequencies in T1 plants generated from the same T0 plant, with or without Cas9—that is V467 (+Cas9, 5.18% NHEJ) and V468 (−Cas9, 5.17% NHEJ), both derived from T0 plant #20 (Table S2).

In general, sgAlpha‐2 was more effective than sgAlpha‐1. It should be noted that lower regeneration was observed in transgenic plants containing sgAlpha1 (0.3% transformation frequency) than plants with sgAlpha2 (1% transformation frequency). Therefore, a possible toxic effect might be also affecting the NHEJ activity of sgAlpha‐1, favouring the regeneration of those lines in which the level of expression of sgAlpha1 is lower, and consequently the mutation frequency. The highest mutation frequencies (62.3%–75.1%) were observed in the BW208‐derived lines transformed with sgAlpha‐2 (Table S2). Three of these T1 lines (T544, T545 and T553) had insertions and deletions (indels) at the target site of between +36 and +158 bp and −1 and −126 bp, respectively (Figure 1b–d). Line T545 had the highest mutation frequency of all analysed lines: ~75% of the sequence reads had indels (Table S2). Transgenic lines of cv DP and cv THA53 showed lower indel frequencies, ranging between 1.50% and 14.77% and 5.16%–7.86%, respectively. Interestingly, the typical −1bp deletion normally observed with CRISPR/Cas9 was very frequent in two of the DP sgAlpha‐2 lines (22.4%–35.6%), but only represented 0.18%–2.9% of the mutations found in the BW208 sgAlpha‐2 lines (Figures 1 and S4). The −1bp deletion was not found in the THA53 lines or in the sgAlpha‐1 lines. The +1 bp insertion, also reported as a typical mutation of CRISPR/Cas9, was only found at low frequency in one of the sgAlpha‐1 lines. A possible explanation for this observation is the preference of certain types of deletions due to microhomology‐mediated repair. The target sites of sgAlpha‐1 and sgAlpha‐2 are highly repetitive, and as shown in Figure S7, repeats between 3 and 36 bp are commonly found flanking the targeted break. The repeats could explain the bias in favour of some of the most frequent mutations observed, such as the −75 and −11 bp deletions in BW208 sgAlpha‐2 lines, the −15 bp deletion in DP sgAlpha‐2 lines and the −36 bp deletion in all BW208, TAH53 and DP lines. DNA insertions represented up to 19% of the total indels (Line T544, Table S2), and they were found to be either fragments of the transformation vectors or other α‐gliadin genes, probably inserted by microhomology‐mediated repair. These results demonstrate that high mutation frequency and specificity can be achieved using CRISPR/Cas9 to modify complex genomic loci such as the α‐gliadin gene family in bread and durum wheat.

**Figure 2:**
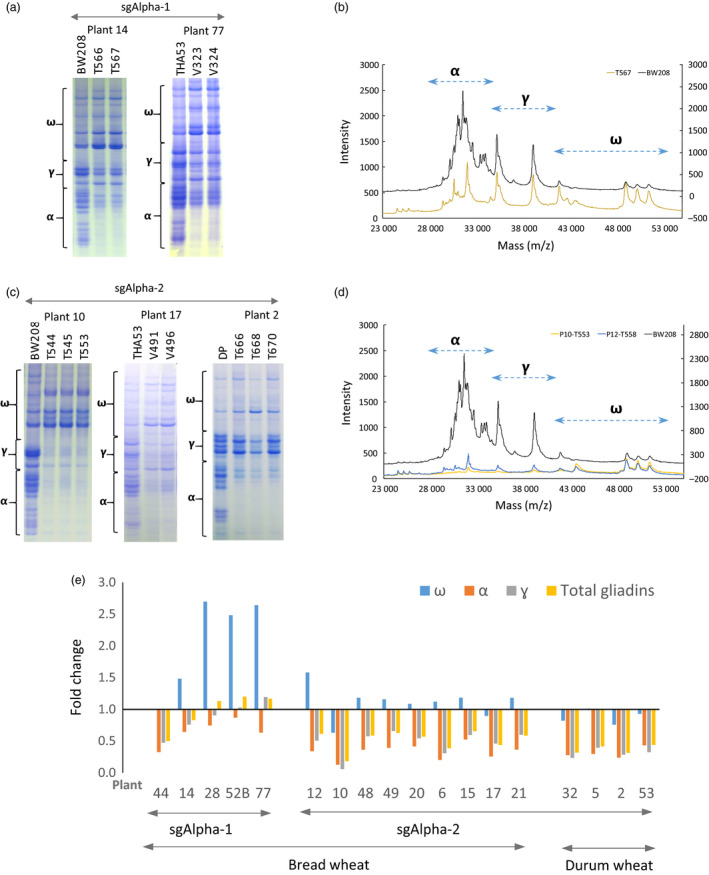
Characterization of sgAlpha‐1 and sgAlpha‐2 mutant plants. (a) A‐PAGE of gliadins from sg Alpha‐1 T1 half‐seeds (named as T566 and T567 lines) derived from T0 plant 14, and V323 and V343 (from T0 plant 77) and the corresponding wild‐type lines BW208 and THA53. Migration of α‐, γ‐, ω‐gliadin protein bands are outlined by brackets (b) MALDI‐TOF analysis of the same gliadin extract in (a) from T567 track and the BW208 wild type. Values are in absolute intensity. Left axis corresponds to T567 and the right axis to the BW208 line. The corresponding range of masses (m/z) for α‐, γ‐, ω‐gliadins are indicated by arrows. (c) A‐PAGE of gliadins from sgAlpha‐2 T1 half‐seeds (named as T544, T545 and T553 lines) from T0 plant 10, V491 and V496 (plant 17), T666, T668 and T670 (plant 2) and the wild‐type lines BW208, THA53 and DP. (d) MALDI‐TOF analysis of the same gliadin extracts in (a) from T553 track (plant 10) and T558 (plant 12, Figure S8), and the BW208 wild type. (e) Bar graph of fold change of α‐, γ‐, ω‐ and total gliadin fractions in bread and durum wheat transformed with sgAlpha‐1 and sgAlpha‐2. Values for each plant were normalized by values of the corresponding wild‐type lines. Note that A‐PAGE analysis is not a quantitative test, and intensity differences observed in the gels might be explained in part by differences in the amount of protein loaded and/or by differences in the staining/distaining process.

To assess the impact of the observed mutations on seed protein composition, gliadin and glutenin content in T1 half‐seeds was qualitatively assessed by A‐PAGE and SDS‐PAGE, respectively (Figures 2a,c and S8). In A‐PAGE and SDS‐PAGE gels, some tracks are not contiguous and have been combined from different, or the same gels (white lines) for presentation clarity. A‐PAGE demonstrated that α‐gliadins were strongly reduced in some of the bread and durum wheat T1 lines (e.g. plants # 6, 10, 12, 15, 17, 32 and 50), and partially reduced in others (e.g. plants # 14, 21, 28 and 48). The γ‐ and ω‐gliadins were also strongly decreased in some lines (e.g. plants # 6, 10, 12, 15 and 21). Additional, novel bands, especially in the region of the gel corresponding to the α‐gliadins, were clearly visible in the A‐PAGE gels, perhaps due to truncation of α‐gliadin coding sequences by mutation (Figure S8). In addition, an accumulation of one mid‐range ω‐gliadin was observed in Plant 10 (Figures 2a and S8). Mass spectrometry (MALDI‐TOF) confirmed the sharp reduction of α‐gliadins in both sgAlpha‐1 and sgAlpha‐2 lines, with the sgAlpha‐2 lines showing a greater reduction in the number of visible peaks (Figure 2b,d). As suggested by the Illumina sequencing results, sgAlpha‐2 more effective reduced the α‐gliadin content, particularly in the BW208 bread wheat lines. The glutenin profile for all lines was comparable to that of the wild type; however, differences in the intensity of the two glutenin fractions were observed, suggesting a mechanism for compensatory reduction in the abundance of these proteins in response to the reduction of α‐gliadins (Figure S8).

Encouraged by these results, HPLC analysis was performed to accurately quantify and characterize the different groups of gliadins and glutenins (Figure 2, Table S3). As expected, α‐gliadin content was significantly reduced in most of the transgenic lines compared to the wild type (32%–82% reduction), especially in the bread and durum wheat lines transformed with sgAlpha‐2. The γ‐gliadins were also significantly reduced by 25%–94% in 15 of the 18 T1 lines analysed, whereas the ω‐gliadins showed the greatest variability: ω‐gliadins were not affected in all four durum wheat lines, significantly up‐regulated (twofold–threefold) in all four bread wheat sgAlpha‐1 T1 lines (Figure 2e), and down‐regulated by 33% in bread wheat Plant 10. Interestingly, this line had the highest reduction in α‐gliadins (82%) and γ‐gliadins (92%) and consequently showed the highest overall gliadin reduction (82%). Amongst the durum wheat lines, Plant 2 had the highest overall gliadin reduction (69%). The reduction in the gliadin content promoted a compensatory effect in glutenins, increasing the HMW fraction, especially in the BW208 and THA53 bread wheat lines (Table S3). The LMW fraction was significantly reduced only in Plant 10 and 32. Similar compensatory effects were observed previously (Gil‐Humanes *et al*., 2011) in wheat lines in which the α‐, γ‐ and ω‐gliadins were down‐regulated by RNAi. In those RNAi lines, compensatory effects provided wheat lines with no difference in the total protein content; however, changes in seed protein expression had important implications on the properties of the flour (Gil‐Humanes *et al*., 2014b), as higher glutenin contents, particularly HMWs, are usually associated with stronger flours. The lines reported here show reduced total gliadin content (specifically the α‐gliadins containing the 33‐mer epitope), increased HMW glutenins and lower gli/glu ratios than the wild type.

**Figure 3:**
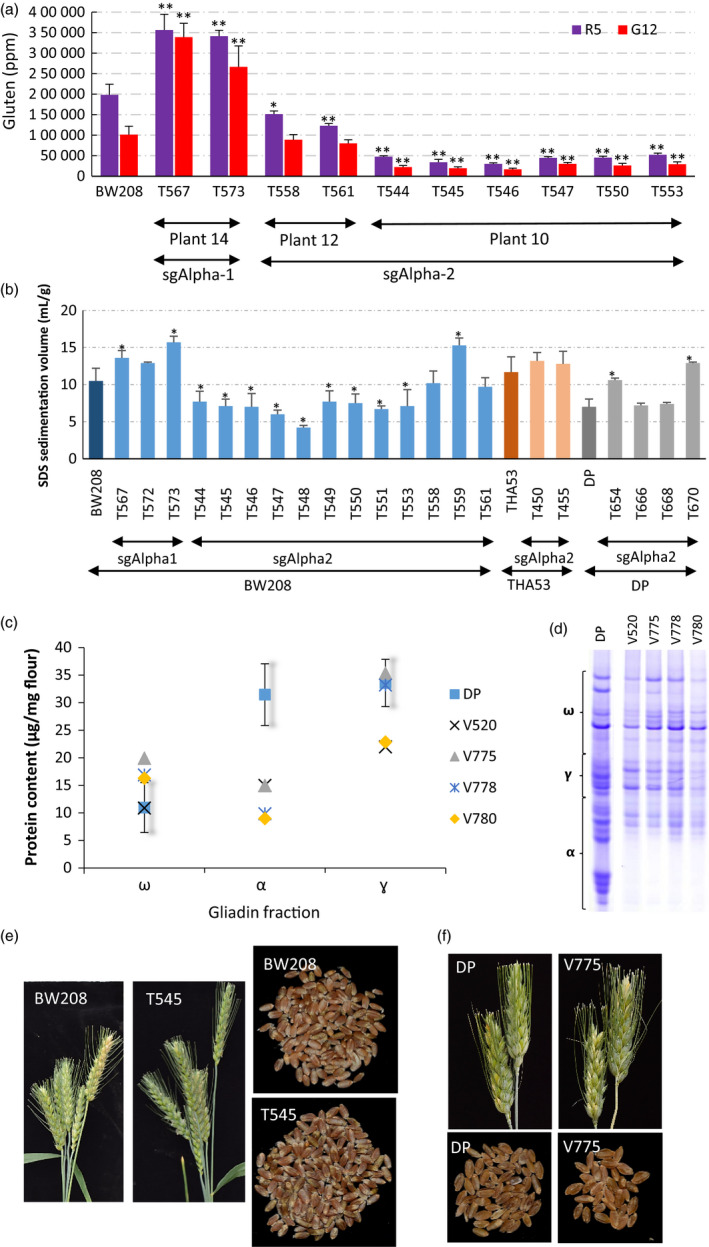
Analysis of Immune reactivity, SDS sedimentation volumes and gliadin profile of nontransgenic DP‐derived lines, and phenotype of sgAlpha‐derived lines. (a) Analysis of T2 seeds of the sgAlpha‐1 and sgAlpha‐2 mutant lines with the monoclonal antibodies (mAb) R5 and G12. Error bars, mean ± SD. Statistically significant differences between each mutant line and the wild type were denoted *P <* *0.05, **P <* *0.01 (Tukey HSD all‐pairwise comparisons test) (b) Sodium dodecyl sulphate (SDS) sedimentation test expressed as mLg‐1. T2 and T3 seeds from each line were bulked and three independent biological replications analysed. Error bars, mean ± SD. * Means are significantly different to wild types as determined by Dunnett's multiple comparisons at *P *<* *0.05. (c) Content of the omega, alpha and gamma‐gliadin fraction of the nontransgenic DP‐derived lines. Error bars, 5% Confidence Interval of the mean value of the wild‐type DP line. (d) A‐PAGE of gliadins from half‐seeds of the nontransgenic DP‐derived lines. Migration of α‐, γ‐, ω‐gliadin protein bands are outlined by brackets. (e) Spikes and seeds of sgAlpha‐2 BW208 mutant line in comparison with its wild type. (f) Spikes and seeds of sgAlpha‐2 DP mutant line in comparison with its wild type.

To confirm that the altered gliadin content effectively reduced the immune reactivity of the flour, we analysed the T2 seeds of the mutant lines with the monoclonal antibodies (mAb) R5 and G12 (Figure 3). R5 is the mAb of choice in the food industry to quantify gluten content and detects a conserved domain (QQPFP) found in most gliadins (not only the ones that are immune reactive) (Valdés *et al*., 2003). The G12 mAb is more specific for detecting reactive epitopes, as it was developed against the 33‐mer peptide (Morón *et al*., 2008). ELISA tests with both mAbs showed a strong reduction in gluten content in the sgAlpha‐2‐derived lines compared to that of the BW208 wild type. In those lines, we observed up to 85% reduction in gluten content (Line T546), and an average reduction of 66.7% and 61.7%, respectively, with the R5 and G12 mAbs. However, both mAbs revealed an increase in the gluten content for lines with sgAlpha‐1. These two lines have a higher ω‐gliadin content—a consequence of the knock‐down of the α‐gliadins (Tables S3 and S4)—which could explain the observed increment in gluten content. Similar increases in gluten content when only the γ‐gliadins were down‐regulated by RNAi were previously reported (Gil‐Humanes *et al*., 2008). In total, these results demonstrate that gluten immunoreactivity can be significantly reduced by editing the α‐gliadin genes containing the immunodominant 33‐mer epitope.

Once we had demonstrated the high efficiency of CRISPR/Cas9 to simultaneously mutate most of the α‐gliadin genes, we next asked whether off‐target mutations were occurring at other sites due to sgAlpha‐1‐ and sgAlpha‐2‐mediated cleavage. First, we looked for possible off‐target mutations in the γ‐ and ω‐gliadin genes, as these proteins were reduced in the mutant lines. Sanger sequencing of fifty‐seven clones containing γ‐gliadin genes (Figure S9) and forty‐three clones with ω‐gliadins genes ─ twenty‐four ω1,2‐gliadins (Figure S10) and nineteen ω5‐gliadins (Figure S11)—showed no off‐target mutations. These results were confirmed by *in silico* search of the sgAlpha‐1 and sgAlpha‐2 sequences (NGG PAM plus 12 nt seed sequence, allowing for up to 2 mismatches) in the wheat prolamin genes annotated in the GenBank (Figure S12a). Additional sequencing of 11–16 clones of amplified LMW from 3 T1 mutant lines (T544, T545 and T553) showed no mutation in the only potential target site identified (Figure S12b). We therefore concluded that the observed decrease in the γ‐ and ω‐gliadins and the glutenins in the mutant lines was not a consequence of off‐target mutations. Rather, we speculate that antisense α‐gliadin sequences could be expressed, resulting in the observed broad reduction in α‐gliadin, as well as the down‐regulation of the other gliadin proteins. Antisense sequences of α‐gliadins could originate in the mutant lines as a consequence of cleavage at two target sites and inversion of the intervening DNA sequence. We tried to detect such hypothetical inversions by predicting the inversion product and performing PCR assays (data not shown); however, none were detected in any of the tested lines.

Next, we expanded our search for off‐target sites to the entire bread wheat genome (Figure S12c). Amongst all potential off‐target sites (41 for sgAlpha1 and 50 for sgAlpha2), only four were annotated genes: a putative MADS box transcription factor (Traes_7BL_F621D9B9E), two genes with unknown function (Traes_2AS_D659E88E9.1, Traes_2AS_8FCC59363.1) and one gene with homology with α‐gliadins (Traes_4AL_4FF5B8837). No mutations were identified in any of these genes in approximately 10 clones sequenced from each gene in the T1 mutant lines T544 (Figure S12d), T545 and T553. Collectively, these results demonstrate the high specificity of the sgRNAs designed to target the α‐gliadins. Further characterization of potential off‐target sites in other nonannotated genes in the genome would be necessary to confirm the lack of undesired mutations.

We next examined whether the mutations were transmitted to the next (T2) generation. Illumina sequencing of 29 T2 plants, with and without Cas9, showed heritability of the mutations (Table S4). Confirming our observations in the T1 generation, the presence/absence of the Cas9 expression vector in T2 plants did not affect the mutation frequencies (Table S4), and we believe that the variability observed between different lines can be explained by (i) stable and somatic mutagenesis due to activity of Cas9 and (ii) segregation of heterozygous stable mutations produced in the previous generations. T2 lines derived from T0 plant #10 were selected for sequencing because they had the least amount of integrated DNA at the cut sites.

The phenotype observed in the prolamin (gliadins and glutenins) content was also inherited, as assessed by evaluating 25 different T2 lines (Table S5), and 16 T3 lines (Table S6) by RP‐HPLC. This demonstrated that the low‐gluten trait is stable and heritable, and will enable its introgression into elite wheat varieties. As observed in T1 seeds, the HMW glutenins were also increased in T2 and T3 seeds of mutant lines (Tables S5 and S6). The HMW fraction is a major determinant of the functionality of wheat flour. We assessed the bread‐making quality of the mutant lines using the SDS sedimentation test by bulking T2 and T3 seeds from each line (Figure 3b). Although some mutant lines showed higher SDS values (higher quality) than the wild‐type control, we observed significant reductions in the SDS values (lower quality) in the mutant lines with the greatest reduction of gliadins. However, in most cases, SDS values in the sgAlpha2 lines were comparable to those of some RNAi lines previously reported showing 97% reduction in the gluten content (Gil‐Humanes *et al*., 2010). Flour from those low‐gluten RNAi lines showed increased stability and better tolerance to over‐mixing (Gil‐Humanes *et al*., 2014b) and allowed the production of bread with baking and sensory properties comparable to those of normal wheat flour (Gil‐Humanes *et al*., 2014a). Consequently, one might expect that mutant lines reported here will produce flour of a good quality and bread‐making performance.

Finally, we tested whether any of the low‐gluten wheat lines we generated were transgene‐ and insertion‐free (i.e. lacked insertions at the cleavage site). We screened the T1 and T2 wheat lines by PCR and Illumina high‐throughput sequencing for the presence of plasmid DNA (Figure S14). Three bread wheat (BW208) and six durum wheat (DP) T2 plants were identified as transgene‐free and insertion‐free (Figure S14). These nontransgenic lines showed reduction in α‐gliadins (Figures 3c,d, and S15) showing. In all cases, all T0, T1 and T2 generations of sgAlpha‐1 and sgAlpha‐2 mutant bread and durum wheat were fully fertile and set seeds, and had normal chromosome numbers (Figure 3c).

We modified the coeliac disease‐causing α‐gliadin gene array using CRISPR/Cas9 technology to obtain nontransgenic, low‐gluten wheat lines. Because of the complexity of the *Gli‐*2 locus and the high copy number of the α‐gliadin genes, traditional plant breeding and mutagenesis have failed to achieve low‐gluten wheat. However, CRISPR/Cas9 efficiently and precisely targeted conserved regions of the α‐gliadin genes in both bread and durum wheat, leading to high‐frequency mutagenesis in most gene copies. Immunoreactivity of the CRISPR‐edited wheat lines was reduced by 85%, as revealed the R5 and G12 ELISA tests. We previously reported the down‐regulation of gliadin genes by RNAi (Gil‐Humanes *et al*., 2010) (Barro *et al*., 2016). Both CRIPSR/Cas9 and RNAi are highly effective for obtaining wheat lines lacking coeliac disease epitopes. However, the main advantages of the CRISPR knockouts vs RNAi are that (i) CRISPR knockouts are stable and heritable mutations that do not involve the expression of a transgene, and (ii) therefore, they provide a phenotype that is independent of environmental conditions. In addition, CRISPR/cas9 would allow different strategies to that reported in this work, that is cutting larger chromosome fragments containing gliadin genes, or even more, replacement of highly immunogenic fragments with others less toxic, keeping the gliadins functionality. In contrast, to obtain all gliadin genes mutated by CRISPR/Cas9, subsequent rounds of mutagenizing will be needed using specific sgRNAs to target the remaining gliadin genes. The low‐gluten, transgene‐free wheat lines described here constitute an unprecedented advance, and the resultant lines provide excellent source material for plant breeding programmes to introgress the low‐gluten trait into elite wheat varieties.

## Methods

### sgRNAs design and plasmid construction

CRISPR/Cas9 reagents were cloned into the pANIC‐6E destination vector (Mann *et al*., 2012) downstream the Ubiquitin1 promoter from maize. Two sgRNAs (sgAlpha‐1: GCCACAAGAGCAAGTTCCAT and sgAlpha‐2: GGTTGTGATGGAAATGGTTG) were designed to recognize conserved regions in the coding sequence of α‐gliadins in hexaploid wheat. To synthesize the expression vectors pANIC‐CR‐Alpha1 and pANIC‐CR‐Alpha2, two Gateway‐compatible donor vectors, one containing TaCas9 (pGdonor‐TaCas9) and another containing the sgRNA (pGdonor‐sgAlpha1 or pGdonor‐sgAlpha2), were combined with pANIC‐6E in a multisite Gateway recombination reaction (Figure S13). pGdonor‐TaCas9 contained a wheat‐codon optimized Cas9 sequence (TaCas9), with an N‐ and C‐terminal nuclear localization signals (NLS) from the simian vacuolating virus 40 (SV40) and nucleoplasmin, respectively, and the OCS terminator sequence. pGdonor‐sgAlpha contained the *Triticum aestivum* U6 RNA polymerase III promoter (TaU6) for expression of the sgRNA, followed by the gRNA sequence (Figure S13).

### Plant material and genetic transformation

Transgenic lines were produced using immature scutella as explants for genetic transformation as described previously (Piston *et al*., 2009). Two bread wheat lines, denoted BW208 and THA53, and one durum wheat line, cv Don Pedro (DP), were used as sources for scutellum isolation and *in vitro* culture. Plasmids carrying the sgRNAs were precipitated onto 0.6‐μm gold particles at 0.75 pmol/mg gold. Regeneration medium was supplemented with 2 mg/L of PPT for selecting transgenic plants. Putative transgenic plants were then transferred to soil and grown to maturity in the greenhouse, and the presence of transformation vectors was confirmed by PCR (Table S1).

### Polyacrylamide gel electrophoresis analysis

Between 6 and 12 mature wheat grains per line were crushed into a fine powder and used to extract sequentially the endosperm storage proteins. Gliadins and glutenins were then separated in A‐PAGE and SDS‐PAGE gels as described (Gil‐Humanes *et al*., 2012).

### Reversed‐phase high‐performance liquid chromatography (RP‐HPLC)

Gliadins and glutenins were extracted and quantified by RP‐HPLC following the protocol reported (Piston *et al*., 2011). Ten half‐seed biological replications were carried out for each transgenic line and wild type. Protein content was expressed as μg protein/mg flour. For each line, 10 half‐grains were analysed.

### Gluten content determination by competitive ELISA

Gluten content was determined by competitive ELISA assays using two monoclonal antibodies; R5 and G12. Samples for R5 were analysed at Centro Nacional de Biotecnología (CSIC, Campus of Cantoblanco, 28049‐Madrid) as described elsewhere (Valdés *et al*., 2003). Samples for G12 were analysed as described previously (Barro *et al*., 2016). Between three and five biological replications for each line were carried out.

### Sodium dodecyl sulphate (SDS) sedimentation test

The SDS sedimentation volume was determined as described (Williams *et al*., 1986). Between two and four biological replications for each line were carried out.

### DNA extraction and PCR conditions for Illumina amplicon sequencing

The Illumina MiSeq system was used for amplicon sequencing producing 2 × 280 paired‐end reads. PCR amplification was carried out using the forward primer aGli900F1 and the reverse primer 33mer1R2_ok (Table S1) with following conditions: 94 °C for 1 min followed by 30 cycles at 94 °C for 15 s, 62 °C for 45 s and 72 °C for 1 min with final extension at 72 °C for 2 min. For PCR amplification, 5 ng of DNA in a 25 μL volume reaction with the following final concentrations: 1× FastStart buffer, 200 nm forward and reverse primers, 200 μm dNTP mix, 1.25 units of FastStart High Fidelity polymerase (Roche Diagnostics, Mannheim, Germany) was used. Preparation of the Illumina amplicon library and sequencing was carried out at the Unidad de Genómica Cantoblanco of Fundación Parque Científico de Madrid (FPCM, Spain). The range of amplicon lengths was check using the Agilent 2100 Bioanalyzer system (Agilent Technologies, Santa Clara, CA 95051).

### Amplicon sequence clustering

Fifty‐three samples were subjected to amplicon sequencing: six samples corresponded to wild‐type DNA (2 BW208, 2 DP and 2 THA53), and 47 to DNA from transgenic lines (Tables S2 and S4). In total, 33.817 millions of reads were obtained. For clustering, the USEARCH software v8.0.1517 (Edgar, 2010) was used. Merging of paired‐end reads was using the ‐fastq_mergepairs command, and for quality filtering by expected errors ‐fastq_filter (‐fastq_maxee 1) commands were used (Edgar and Flyvbjerg, 2015). Then, all 11.676 millions of cleaned and filtered reads were clustered with ‐cluster_otus mode, 100% homology and ‐search_exact command for mapping and to extract the consensus sequence for each cluster. To extract the high‐confidence amplicon variants for each sample, samples with less than five reads in a given cluster were removed from that cluster. As clustering was at 100%, consensus clusters were considered as unique genes. As samples have different numbers of reads, frequencies were calculated for each sample by dividing the number of reads for a given amplicon gene (n) by the total count for a sample (N). Then, all gene sequences were processed using Geneious version 9.1.4 (Biomatters Ltd., Auckland, New Zealand; available at http://www.gene-ious.com/). First, a reference unique gene library was constructed for each of the wild‐type lines. Genes with different lengths were used as reference sequences for aligning and mapping of amplicon genes from mutant lines. Second, genes present in mutant lines were aligned and mapped to reference gene library constructed previously using the BBmap aligner (https://sourceforge.net/projects/bbmap/). MAFFT software v7.222 (Katoh *et al*., 2002) and FastTree software (Price *et al*., 2010) were used for multiple sequence alignment and maximum‐likelihood phylogenetic trees, respectively, to determine the corresponding nonmutated sequences.

### PCR amplification of γ‐ and ω‐gliadin genes and sequencing by Sanger

The gene‐specific primers for Sanger sequencing of γ‐ and ω‐gliadin genes are in Table S1. These primers amplified from signal peptide in the 5′ to end of the coding region in the 3′. The complete γ‐ and ω‐gliadin genes were amplified by PCR as follow: 94 °C for 4 min followed by 35 cycles at 94 °C for 15 s, 60 °C or 66 °C (γ‐gliadins and ω‐gliadins, respectively) for 1 min and 72 °C for 1 min 30 s, with final extension at 72 °C for 7 min. For PCR amplification, 200 ng of DNA in a 25 μL volume reaction consisting of 400 nm forward and reverse primers, 320 μm dNTP mix, a mixture of 0.013 units Pfu DNA polymerase (Biotools, B&M Labs, Madrid, Spain) and 0.650 units Taq DNA polymerase (Biotools) was used. PCR products were checked by 1% agarose gel electrophoresis.

Full‐length DNA sequences were ligated into pGEM‐T Easy vector (Promega, Madison, WI) and cloned into Escherichia coli DH5α cells. Sequencing was carried out by Stab Vida (Caparica, Portugal). We sequenced 102 clones (35 wild types and 67 mutant lines) and 78 clones (26 wild types and 52 mutant lines) for the γ‐ and ω‐gliadin genes, respectively.

### Detection of *bar* and Cas9 genes, and other DNA plasmid regions

PCR was performed to detect insertions of plasmid DNA using primers listed in Table S1. For detection of *bar* and Cas9 genes, and PVS1 stability (sta) region, Octopine synthase polyA signal, kanamycin resistance gene and *Panicum virgatum* ubiquitin 1 promoter, 300 ng of DNA was used in a 25 μL volume reaction, consisting of 400 nm forward and reverse primers, 320 μm dNTP mix and 0.650 units Taq DNA polymerase (Biotools, Madrid, Spain). PCR conditions were as follows: 94 °C for 4 min followed by 35 cycles at 94 °C for 15 s, 58 °C for 45 s or 30 s for Cas9 and the other genes, respectively, and 72 °C for 1 min 30 s with final extension at 72 °C for 7 min.

Specifics primers (Table S1) were designed to be used with aGli900 Forward primer for the amplification of each insertion detected by deep sequencing. PCR conditions for the amplification of insertions were as followed: 94 °C for 5 min followed by 35 cycles at 94 °C for 30 s, 60 °C for 30 s and 72 °C for 30 s with a final extension at 72 °C for 5 min. For PCR amplification, 300 ng of DNA in a 25 μL volume reaction consisting of 400 nm forward and reverse primers, 320 μm dNTP mix and 0.650 units Taq DNA polymerase (Biotools) was used. All PCR products were checked by 1% agarose gel electrophoresis.

### Analysis of off‐target mutations

Potential off‐targets in the wheat genome were detected by two different methods. First, we performed an *in silico* search of the minimal active sequence of the sgRNAs in the prolamin genes (except α‐gliadins) deposited in the GenBank database (http://www.ncbi.nlm.nih.gov/). We used the seed sequence (12 nt upstream the PAM sequence) of the sgAlpha‐1 and sgAlpha‐2 plus the NGG PAM sequence and searched for homology in 179 γ‐gliadins, 15 ω‐gliadins, 40 HMW glutenins and 239 LMW glutenins, allowing up to two mismatches in the seed sequence. Then, we expanded our *in silico* search for off‐target sites to the whole genome of wheat by searching for perfect matches of the seed sequence (12 nt) plus PAM in the reference genome of bread wheat (http://plants.ensembl.org/index.html).

Potential off‐targeted genes were characterized in 3 T1 mutant plants (T544, T545 and T553). Specific primers were designed to PCR amplify a 267 to 323 bp fragment encompassing the potential off‐target site of sgAlpha‐1 or sgAlpha‐2 in the identified genes (Traes_7BL_F621D9B9E (MADS box transcription factor), Traes_2AS_D659E88E9.1, Traes_2AS_8FCC59363.1 and the gene family of LMW glutenins) (Table S1). Amplicons were cloned, and between 24 and 39 clones were sequenced for each of the genes.

### Matrix‐assisted laser desorption ionization‐time of flight (MALDI‐TOF) analysis

Gliadin fractions were extracted from wheat flours using 60% ethanol for 1 h at room temperature in a rotary shaker and centrifuged at 12000 ***g*** for 5 min at room temperature. Previously, samples were washed twice with 0.5 m NaCl for 30 min at 4 °C in a rotary shaker and centrifuged at 12000 ***g*** for 5 min at 4 °C, to remove albumins/globulins fraction.

The ethanolic supernatants obtained for each sample were diluted at 1 : 1 ratio (v/v) with matrix solution (10 mg/mL 2,5 Dihydroxyacetophenone in 50% aqueous acetonitrile and 100 mM ammonium citrate). A 1.0 μL aliquot of this mixture was manually deposited onto a 386‐well OptiTOF™ Plate (Sciex) and allowed to dry at room temperature. For MALDI‐TOF/TOF analysis, samples were automatically acquired in an ABi 4800 MALDI‐TOF/TOF mass spectrometer (Sciex) in positive ion linear mode (the ion acceleration voltage was 25 kV for MS acquisition). The detection mass range was set between 1500 and 80 000 m/z.

### Statistical analysis

Data were analysed with the statistical software Statistix v10 (Analytical software, PO Box 12185, Tallahassee, FL 32317). The differences in the data were assessed using analysis of the variance (ANOVA), followed by the two‐tailed Dunnett's post hoc test for median multiple comparisons. *P* values lower than 0.05 were considered significant. Shapiro–Wilk normality test was used to verify that data was normally distributed, and logarithmic or Box Cox transformations were applied whenever a variable did not pass the test. Figures were drawn using the Microsoft Excel and PowerPoint software (Microsoft Corporation).

## Conflict of interest

Dr Javier Gil‐Humanes is currently the Crop Pipeline Manager at Calyxt Inc. The authors have declared a conflict of interest.

## Author contributions

F.B. and J.G.H. conceived the project; F.B. and J.G.H. designed experiments; S.S.L., C.V.O. and M.J.G. performed most of the experiments; F.B., J.G.H., S.S.L., C.V.O., M.J.G., D.V. and F.B. analysed experiments; C.S. performed the G12 ELISA assay; F.B., J.G.H. and D.V. wrote the manuscript with assistance from others authors.

## Supporting information


**Figure S1** Nucleotide alignments of the highly represented α‐gliadin genes detected by Illumina sequencing (accounting for nearly 85% of the total reads) in the wild type lines of bread wheat (a) cv BW208, (b) cv TAH53, and (c) durum wheat cv DP.

**Figure S2** Protein alignments of the highly represented α‐gliadin genes in the wild type lines of bread wheat cv BW208 (a) and cv TAH53 (d), and durum wheat cv DP (f).

**Figure S3** Estimated α‐gliadin genes present in the wild type lines and mutated in the mutant lines by sgAlpha‐2.

**Figure S4** Gene editing of α‐gliadins in durum wheat cv
DP.

**Figure S5** Gene editing of α‐gliadins in bread wheat cv TAH53.

**Figure S6** Gene editing of α‐gliadins in bread wheat cv BW208.

**Figure S7** Microhomology‐mediated repair of α‐gliadins targeted with sgAlpha‐ 2.

**Figure S8** Gliadin and glutenin protein fractions analysed by A‐PAGE and SDS‐PAGE from T1 half‐seeds derived from T0 lines transformed with sgAlpha‐1 and sgAlpha‐2 constructs.

**Figure S9** Off‐target mutations detection in γ‐gliadin genes of BW208 wild type and two T1 mutant lines.

**Figure S10** Off‐target mutations detection in ω1,2‐gliadin genes of BW208 wild type and two T1 mutant lines.

**Figure S11** Off‐target mutations detection in ω5‐gliadin genes of BW208 wild type and two T1 mutant lines (T544 and T545).

**Figure S12** Off‐target mutations detection in BW208 mutant lines.

**Figure S13** Multisite Gateway cloning of pANIC6E‐CR‐Alpha1 vector.

**Figure S14** Analysis by PCR and Illumina high‐throughput sequencing for the presence of the plasmid DNA; *bar* and Cas9 genes, PVS1 stability (sta) region, Octopine synthase polyA signal, and *Panicum virgatum* ubiquitin 1 promoter; and insertions in sgAlpha‐2 derived lines.

**Figure S15** Protein analysis of non‐transgenic transgenic (transgene‐free and insertion‐free) lines determined by RP‐HPLC and A‐PAGE gels.

**Table S1** List and sequence of primers for PCR and Illumina sequencing

**Table S2** Illumina sequencing of alpha‐gliadins in 18 T1 bread and durum wheat transgenic lines

**Table S3** Gliadin and glutenin contents, total prolamin content, and gliadin to glutenin ratio of transgenic and wild‐type T1 half‐seeds from T0 lines

**Table S4** Illumina sequencing of alpha‐gliadins in 29 T2 bread and durum wheat transgenic lines

**Table S5** Gliadin and glutenin contents, total prolamin content, gliadin to glutenin ratio, and SDS sedimentation test of transgenic and wild‐type T2 seeds from T1 lines

**Table S6** Gliadin and glutenin contents, total prolamin content, gliadin to glutenin ratio, and SDS sedimentation test of transgenic and wild‐type T3 seeds from T2 lines
