# Supplementary figures and images for: Low‐gluten, nontransgenic wheat engineered with CRISPR/Cas9
Source: Plant Biotechnol J. 2017 Nov 24;16(4):902–10. doi: 10.1111/pbi.12837 (PMC5867031; doi:10.1111/pbi.12837)

## Slide 1
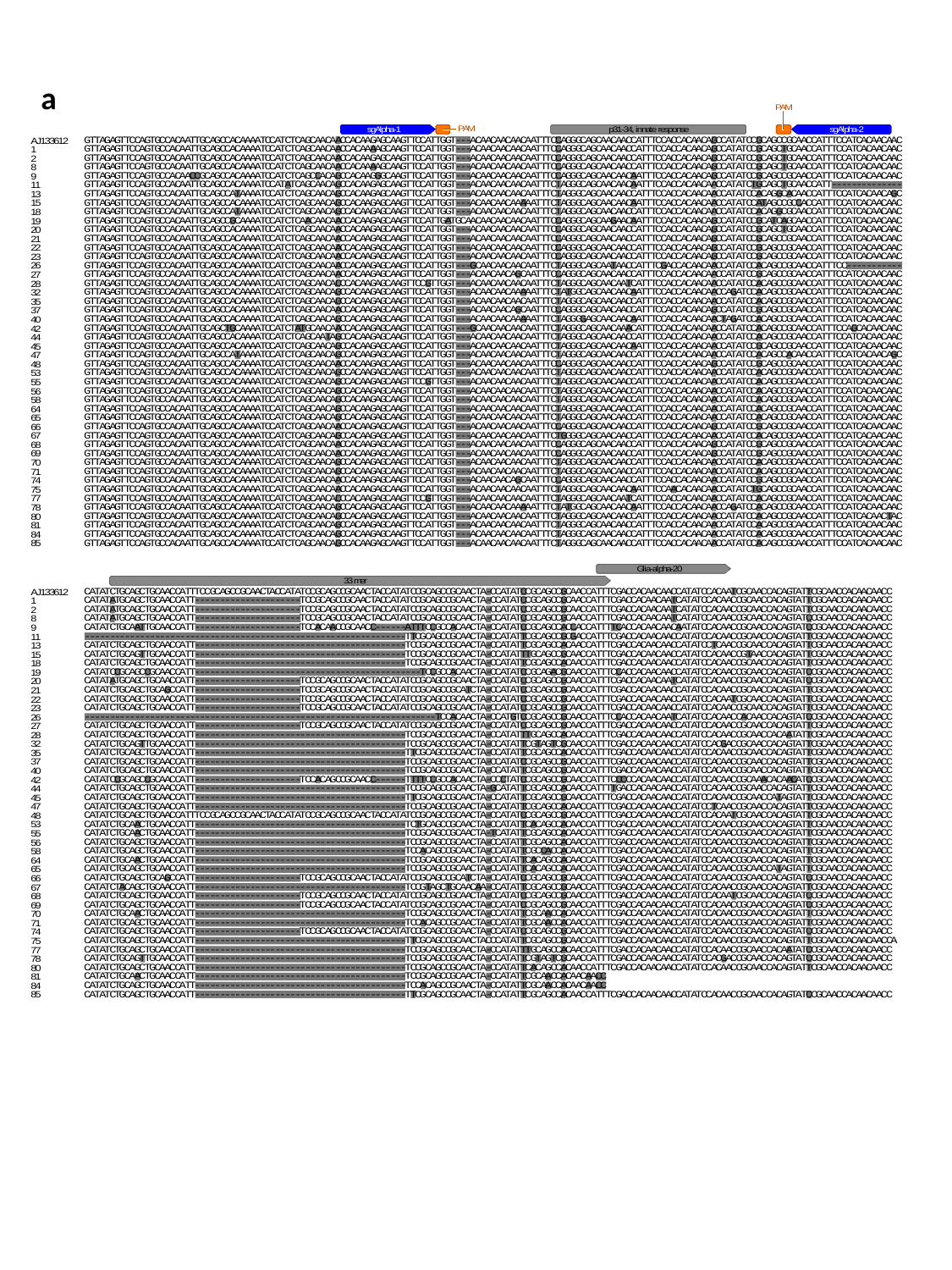

a

## Slide 2
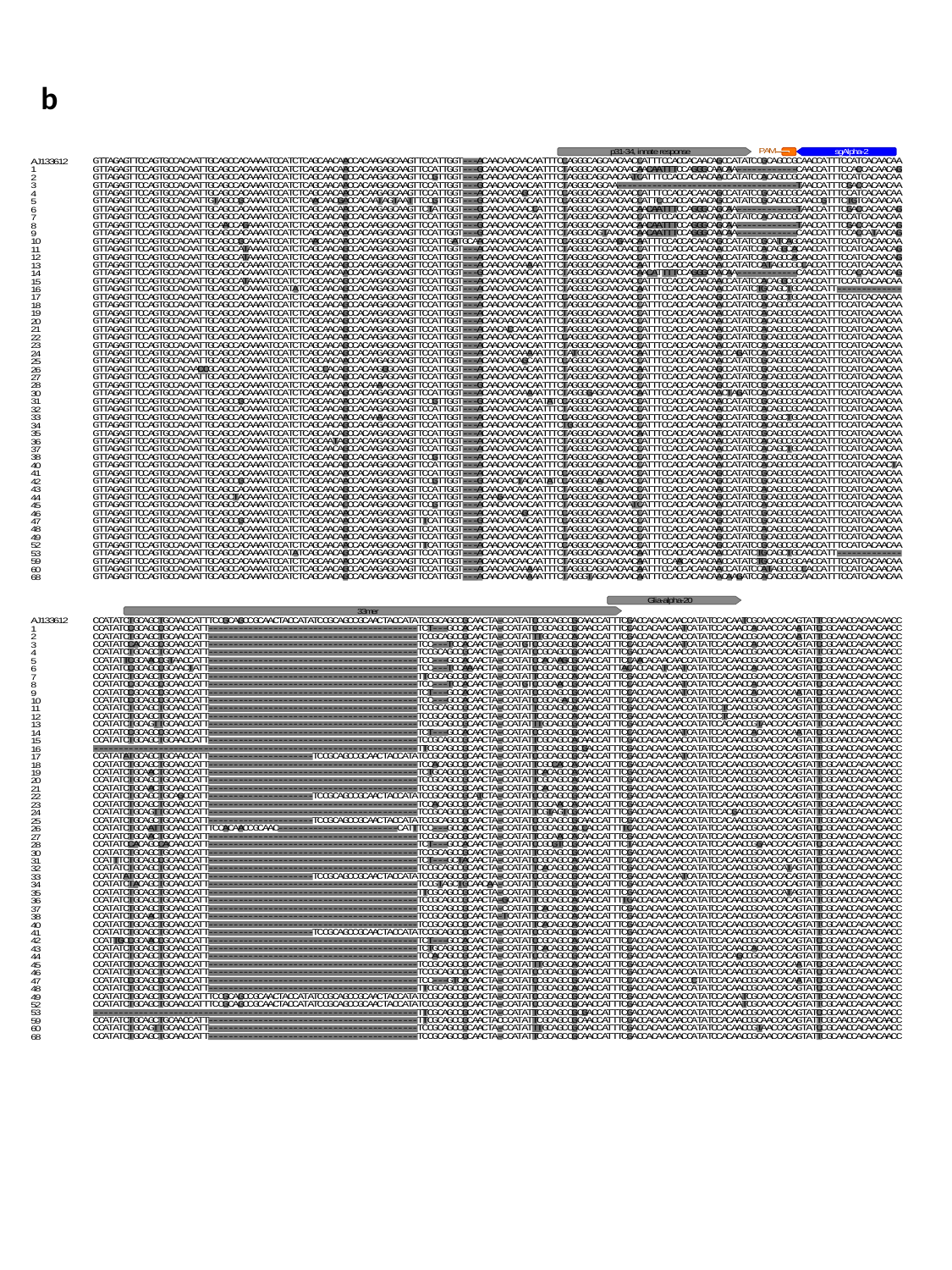

b

## Slide 3
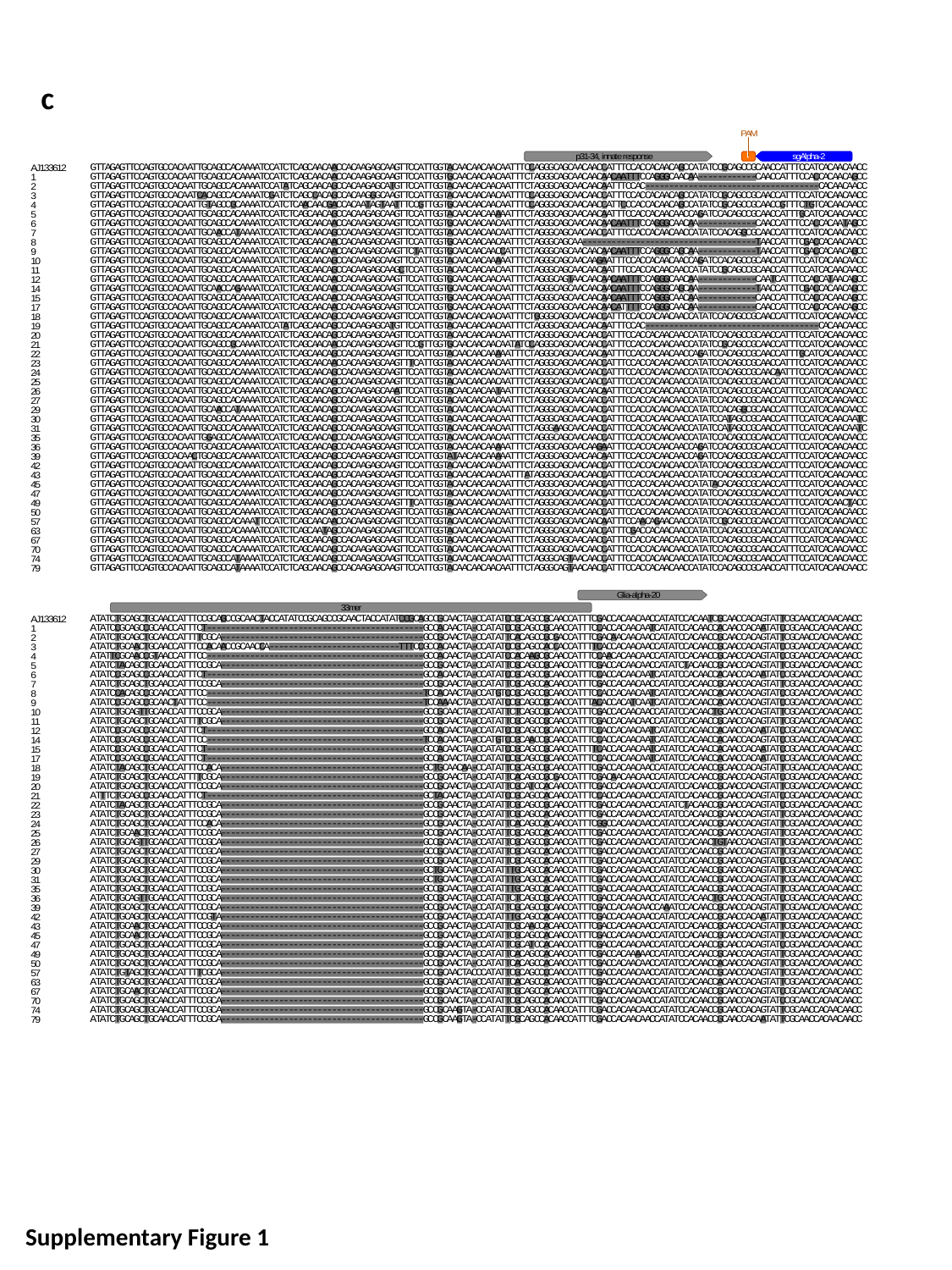

c
Supplementary Figure 1

Supplement: Supplementary file 1 — Figure S1 Nucleotide alignments of the highly represented α‐gliadin genes detected by Illumina sequencing (accounting for nearly 85% of the total reads) in the wild type lines of bread wheat (a) cv BW208, (b) cv TAH53, and (c) durum wheat cv DP. [file PBI-16-902-s020.pptx]

## Slide 1
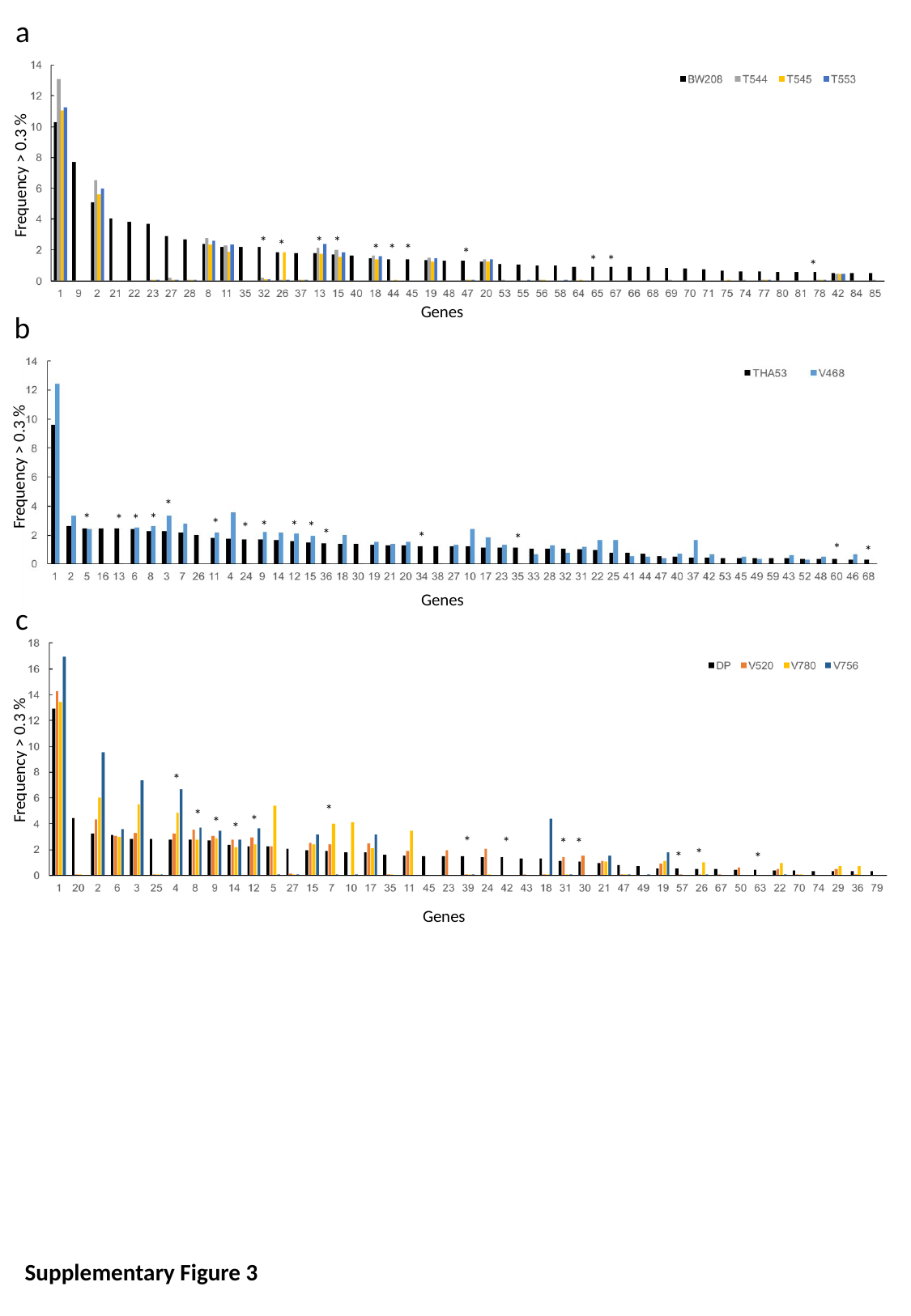

a
Frequency > 0.3 %
*
*
*
*
*
*
*
*
*
*
*
Genes
b
Frequency > 0.3 %
*
*
*
*
*
*
*
*
*
*
*
*
*
*
*
Genes
c
*
*
*
*
*
*
*
*
*
*
*
*
*
Frequency > 0.3 %
Genes
Supplementary Figure 3

Supplement: Supplementary file 3 — Figure S3 Estimated α‐gliadin genes present in the wild type lines and mutated in the mutant lines by sgAlpha‐2. [file PBI-16-902-s019.pptx]

## Slide 1
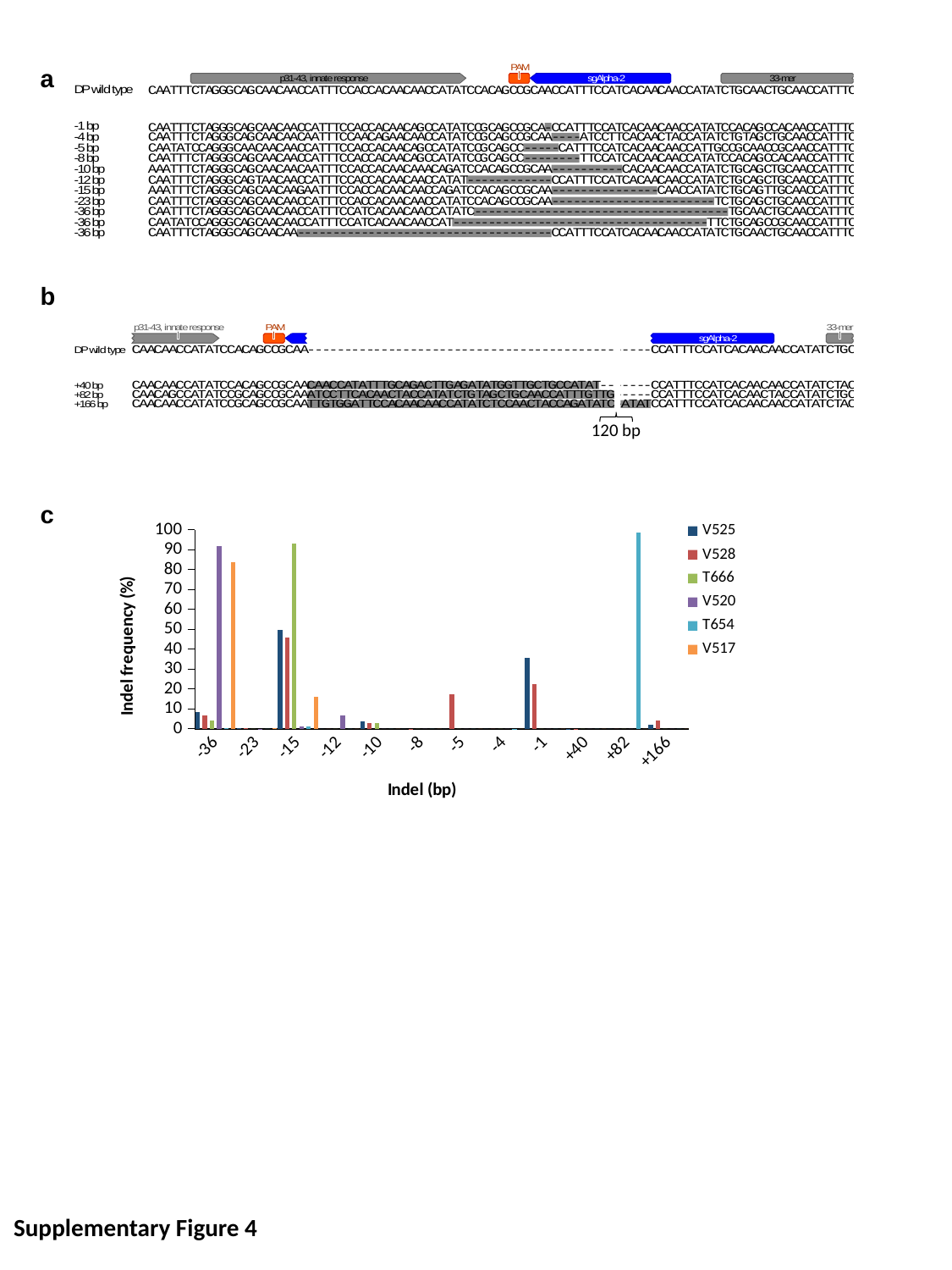

a
b
120 bp
c
### Chart
| Category | V525 | V528 | T666 | V520 | T654 | V517 |
|---|---|---|---|---|---|---|
| -36 | 8.60114681957595 | 6.825191078532151 | 4.000763261671543 | 91.789382026788 | 0.300020691082144 | 83.56870656803405 |
| -23 | 0.386718229097213 | 0.429088633620882 | 0.0 | 0.0153660972673957 | 0.0 | 0.493162967944407 |
| -15 | 49.67328977196959 | 45.84096902516417 | 93.05431878895814 | 1.390631802699311 | 1.22077384647217 | 15.93813046402152 |
| -12 | 0.0 | 0.0 | 0.0 | 6.804620073245064 | 0.0 | 0.0 |
| -10 | 3.738276214606392 | 2.717561346265588 | 2.94491794937031 | 0.0 | 0.0 | 0.0 |
| -8 | 0.0 | 0.102802485138336 | 0.0 | 0.0 | 0.0 | 0.0 |
| -5 | 0.0 | 17.36021096857819 | 0.0 | 0.0 | 0.0 | 0.0 |
| -4 | 0.0 | 0.0 | 0.0 | 0.0 | 0.0569004758948893 | 0.0 |
| -1 | 35.56918700271147 | 22.37965404728914 | 0.0 | 0.0 | 0.0 | 0.0 |
| +40 | 0.0622305196248389 | 0.0312877128681893 | 0.0 | 0.0 | 0.0 | 0.0 |
| +82 | 0.0 | 0.0 | 0.0 | 0.0 | 98.42230498655067 | 0.0 |
| +166 | 1.969151442414544 | 4.313234702543245 | 0.0 | 0.0 | 0.0 | 0.0 |Supplementary Figure 4

Supplement: Supplementary file 4 — Figure S4 Gene editing of α‐gliadins in durum wheat cv DP. [file PBI-16-902-s018.pptx]

## Slide 1
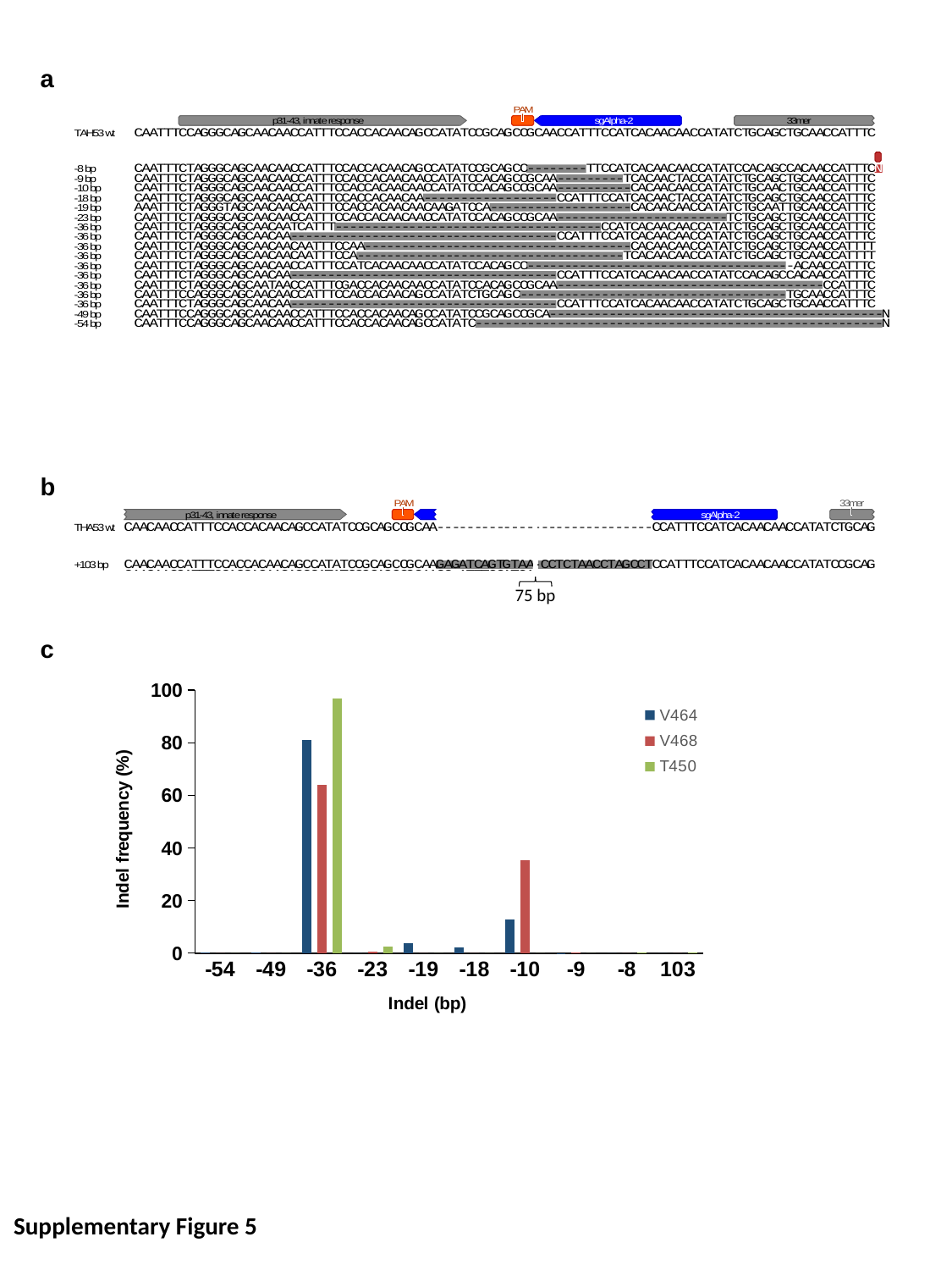

a
b
75 bp
c
### Chart
| Category | V464 | V468 | T450 |
|---|---|---|---|
| -54.0 | 0.105408788457738 | 0.0 | 0.0 |
| -49.0 | 0.191053429079649 | 0.0 | 0.0 |
| -36.0 | 80.861716845642 | 64.13148788927337 | 96.9376979936642 |
| -23.0 | 0.0 | 0.602076124567474 | 2.393523407250968 |
| -19.0 | 3.761776138085513 | 0.0 | 0.0 |
| -18.0 | 2.259700902562751 | 0.0 | 0.0 |
| -10.0 | 12.74787535410765 | 35.16955017301038 | 0.0 |
| -9.0 | 0.0724685420646947 | 0.0968858131487889 | 0.0 |
| -8.0 | 0.0 | 0.0 | 0.351988736360436 |
| 103.0 | 0.0 | 0.0 | 0.316789862724393 |Supplementary Figure 5

Supplement: Supplementary file 5 — Figure S5 Gene editing of α‐gliadins in bread wheat cv TAH53. [file PBI-16-902-s017.pptx]

## Slide 1
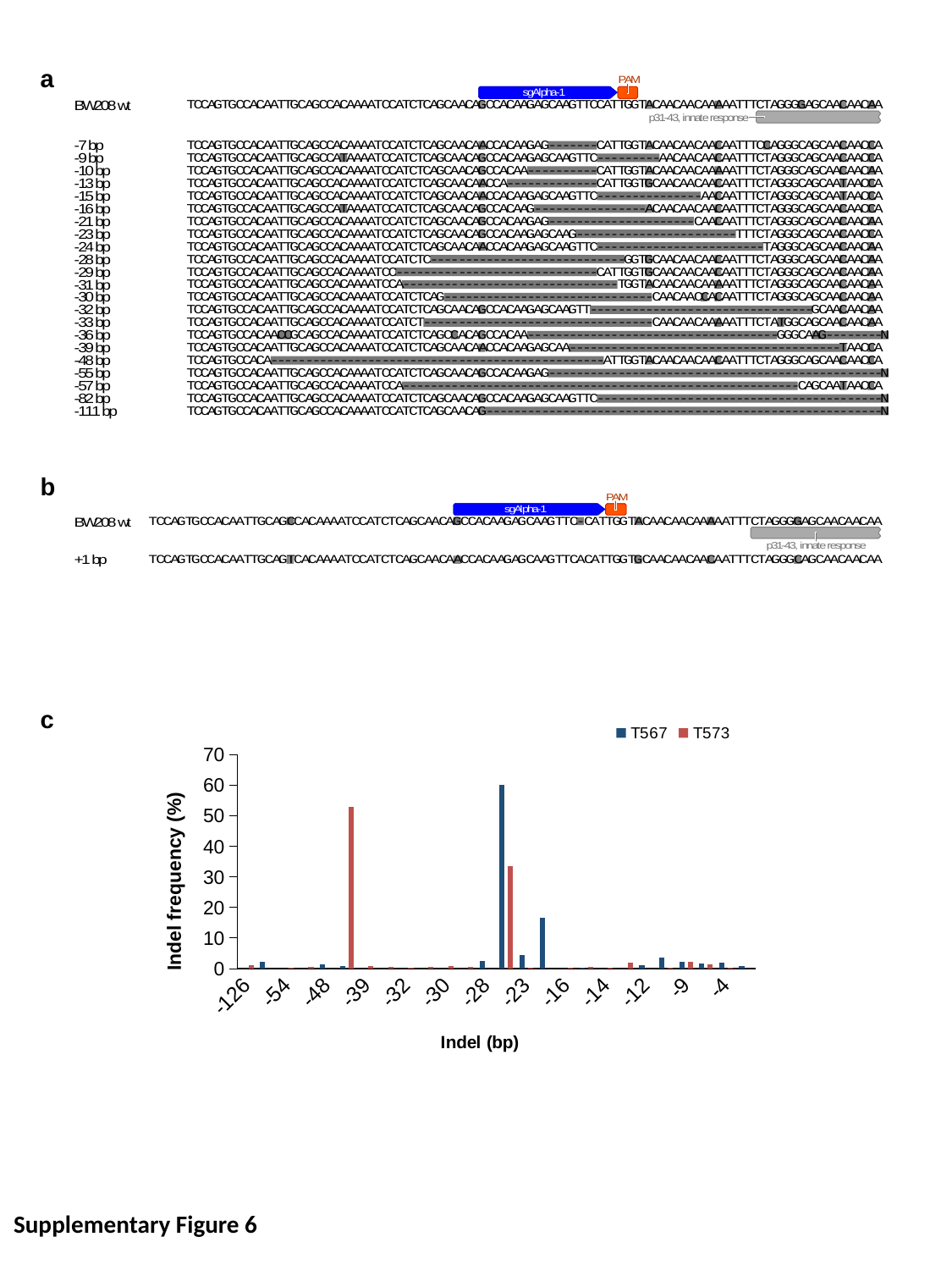

a
b
c
### Chart
| Category | T567 | T573 |
|---|---|---|
| -126 | 0.0 | 1.105249937201708 |
| -79 | 2.362948960302457 | 0.0 |
| -54 | 0.0 | 0.276312484300427 |
| -52 | 0.0 | 0.577744285355438 |
| -48 | 1.417769376181474 | 0.0 |
| -45 | 0.756143667296787 | 52.85104245164512 |
| -39 | 0.0 | 0.954534036674203 |
| -33 | 0.0 | 0.627982918864607 |
| -32 | 0.0 | 0.276312484300427 |
| -31 | 0.0 | 0.70334086912836 |
| -30 | 0.0 | 0.828937452901281 |
| -29 | 0.0 | 0.70334086912836 |
| -28 | 2.646502835538752 | 0.0 |
| -24 | 60.01890359168233 | 33.38357196684242 |
| -23 | 4.44234404536862 | 0.276312484300427 |
| -21 | 16.6351606805293 | 0.0 |
| -16 | 0.0 | 0.276312484300427 |
| -15 | 0.283553875236295 | 0.653102235619191 |
| -14 | 0.0 | 0.376789751318764 |
| -13 | 0.0 | 2.009545340366742 |
| -12 | 1.039697542533081 | 0.0 |
| -10 | 3.591682419659728 | 0.251193167545843 |
| -9 | 2.173913043478261 | 2.336096458176338 |
| -7 | 1.606805293005671 | 1.281085154483798 |
| -4 | 2.079395085066162 | 0.251193167545843 |
| +1 | 0.945179584120983 | 0.0 |Supplementary Figure 6

Supplement: Supplementary file 6 — Figure S6 Gene editing of α‐gliadins in bread wheat cv BW208. [file PBI-16-902-s001.pptx]

## Slide 1
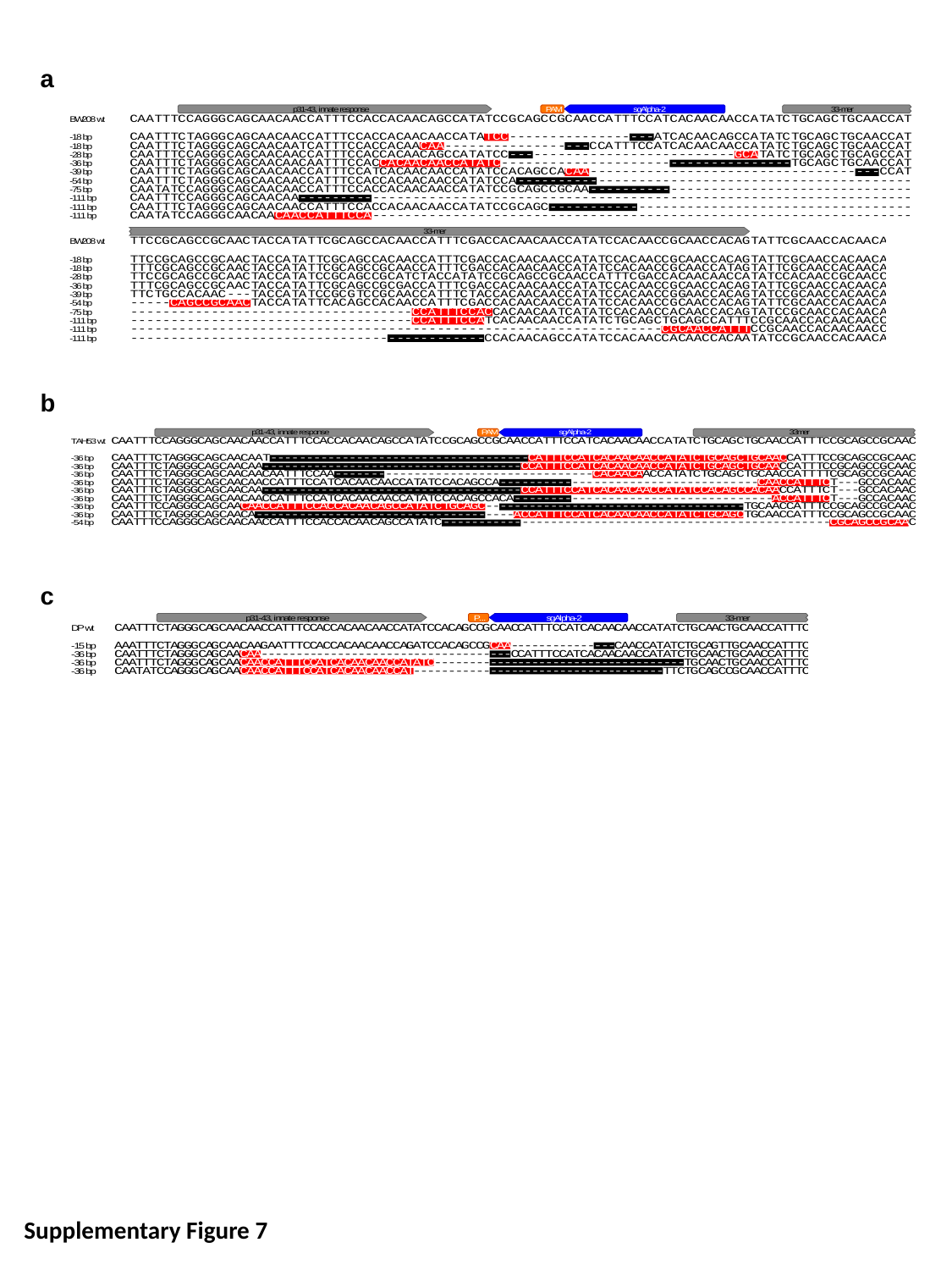

a
b
c
Supplementary Figure 7

Supplement: Supplementary file 7 — Figure S7 Microhomology‐mediated repair of α‐gliadins targeted with sgAlpha‐ 2. [file PBI-16-902-s002.pptx]

## Slide 1
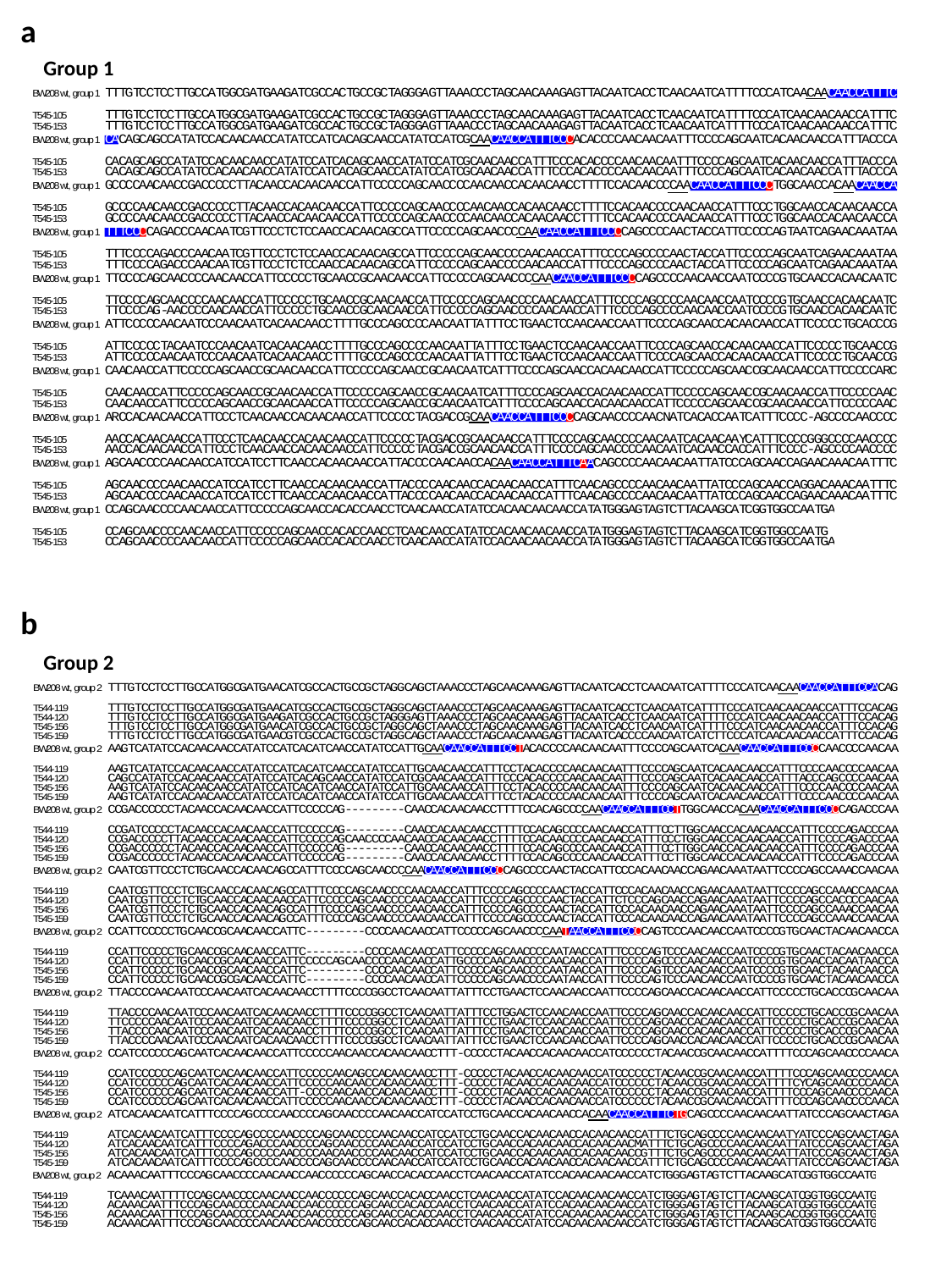

a
Group 1
b
Group 2

## Slide 2
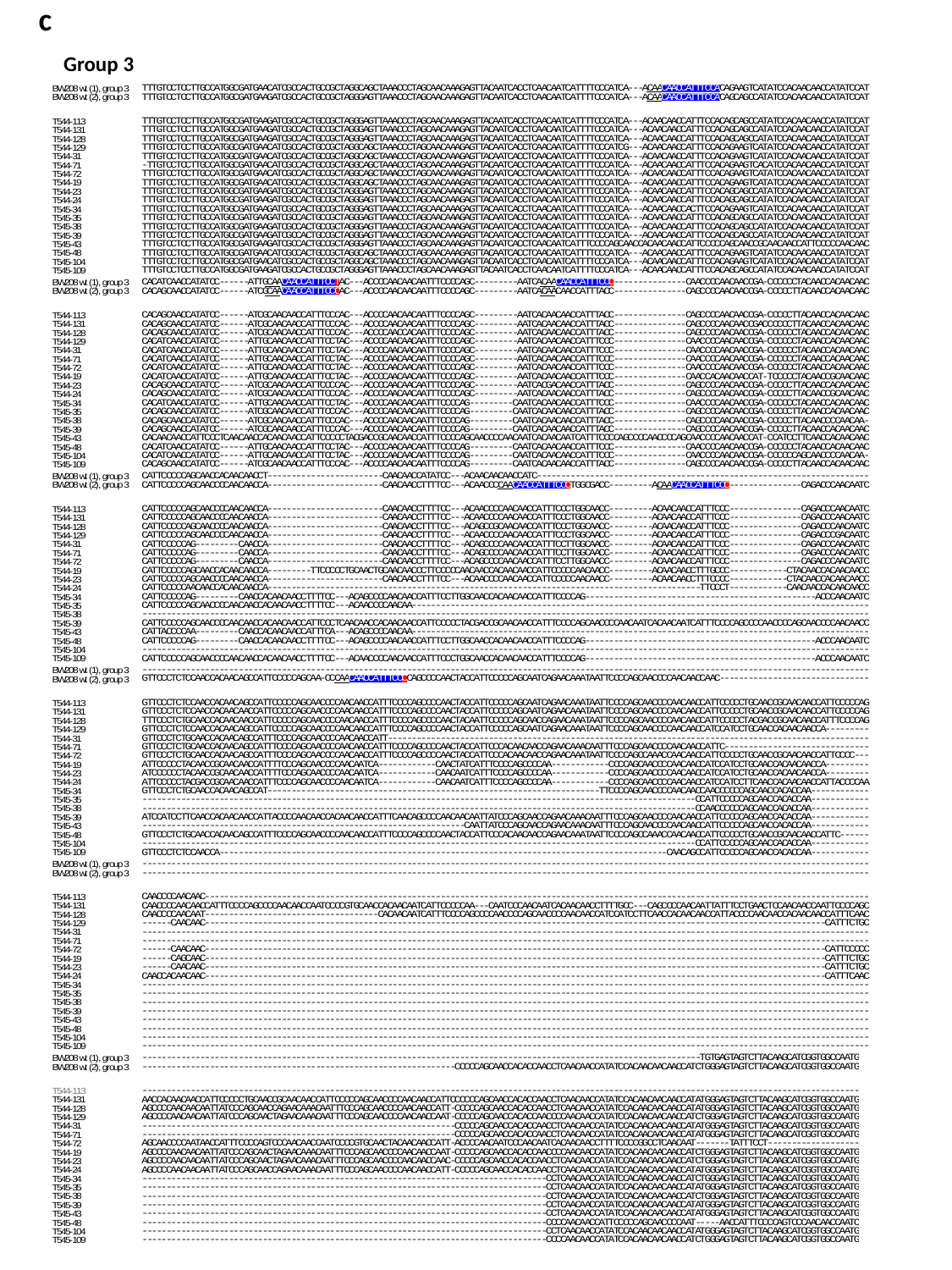

c
Group 3

## Slide 3
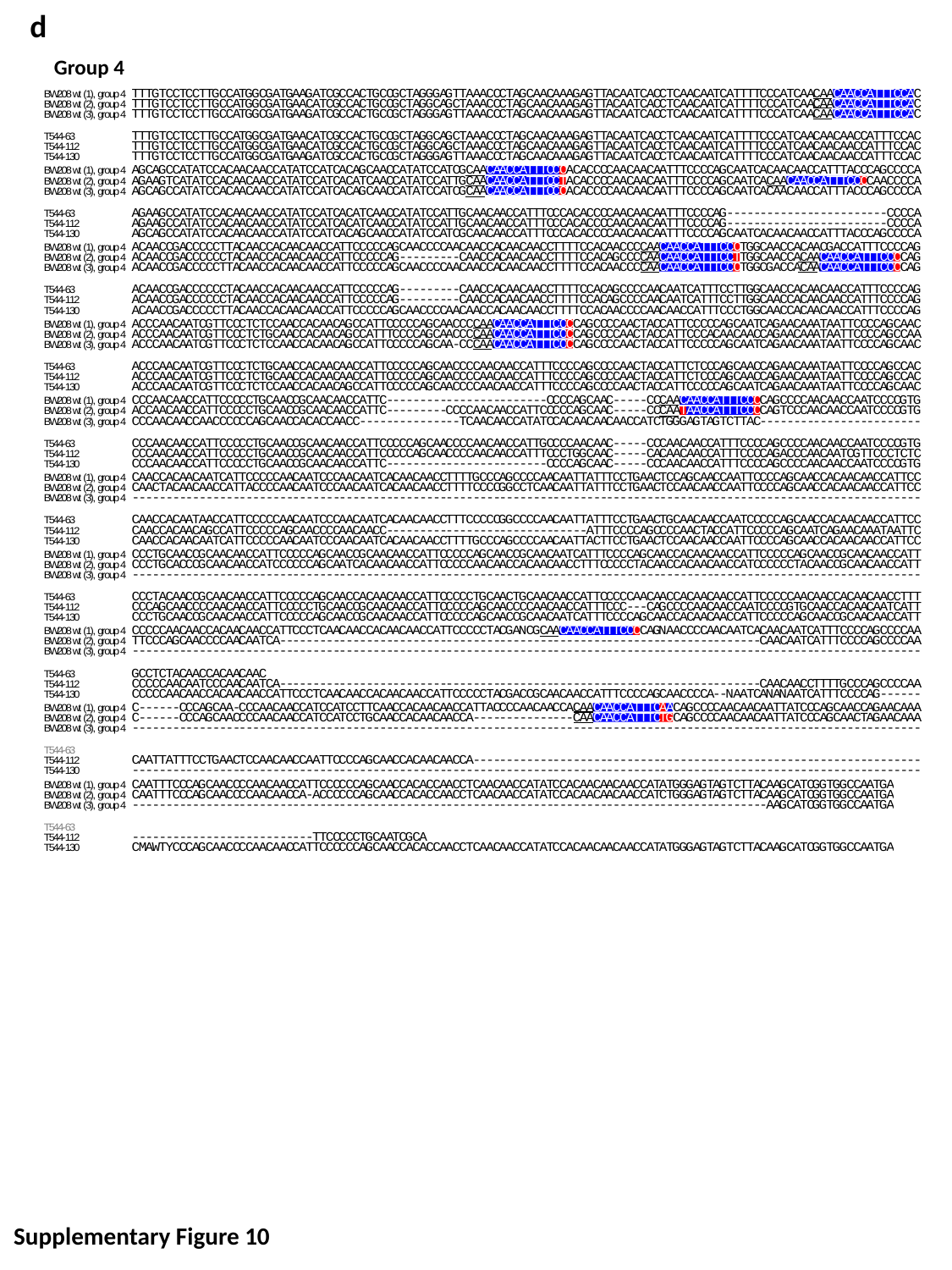

d
Group 4
Supplementary Figure 10

Supplement: Supplementary file 10 — Figure S10 Off‐target mutations detection in ω1,2‐gliadin genes of BW208 wild type and two T1 mutant lines. [file PBI-16-902-s005.pptx]

## Slide 1
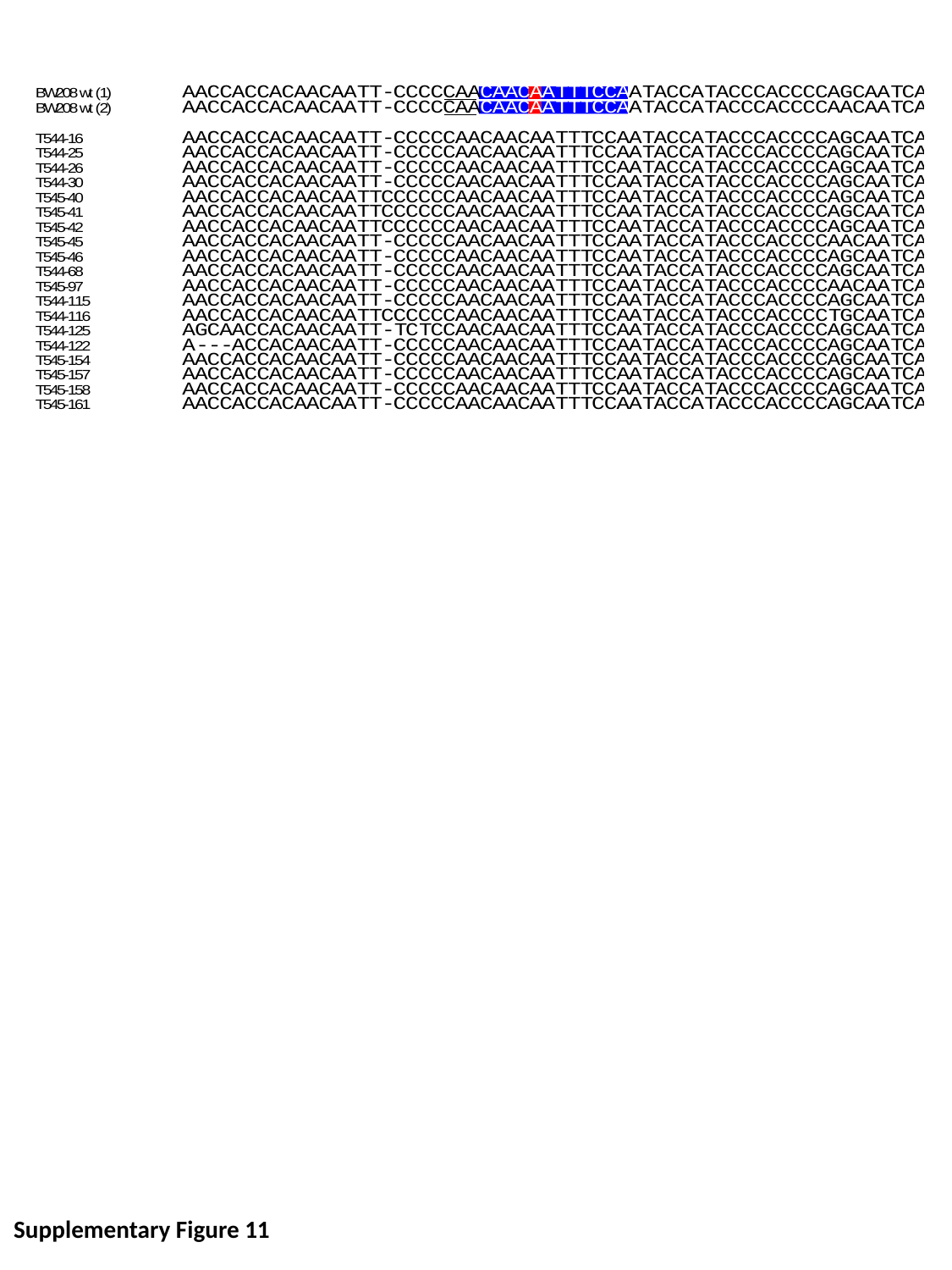

Supplementary Figure 11

Supplement: Supplementary file 11 — Figure S11 Off‐target mutations detection in ω5‐gliadin genes of BW208 wild type and two T1 mutant lines (T544 and T545). [file PBI-16-902-s006.pptx]
