# Supplementary material for: Low‐gluten, nontransgenic wheat engineered with CRISPR/Cas9
Source: Plant Biotechnol J. 2017 Nov 24;16(4):902–10. doi: 10.1111/pbi.12837 (PMC5867031; doi:10.1111/pbi.12837)
Supplement: Supplementary file 2 — Figure S2 Protein alignments of the highly represented α‐gliadin genes in the wild type lines of bread wheat cv BW208 (a) and cv TAH53 (d), and durum wheat cv DP (f). [file PBI-16-902-s021.pptx]

## Slide 1
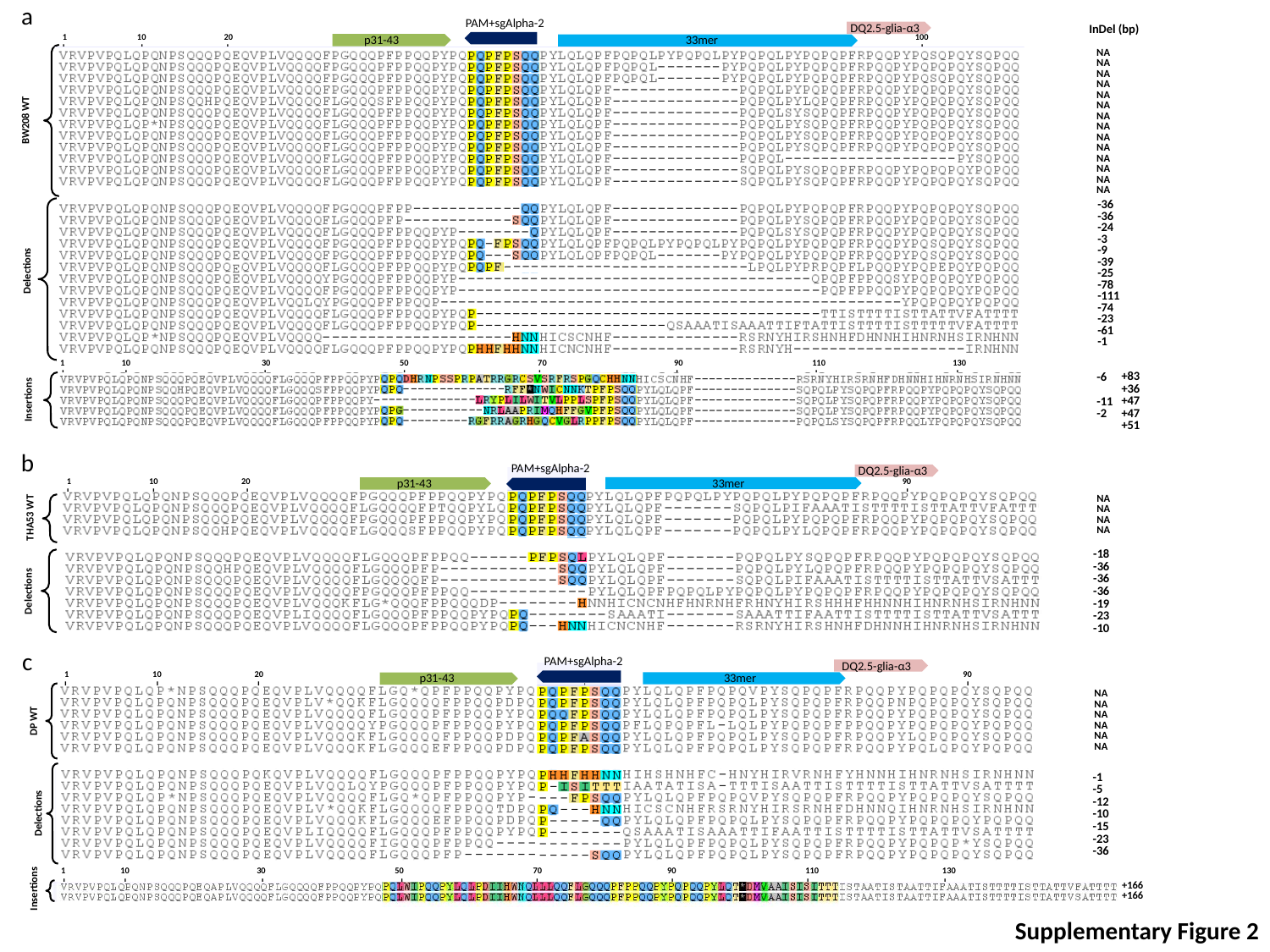

a
InDel (bp)
PAM+sgAlpha-2
DQ2.5-glia-α3
1 10 20 100
I I I I
p31-43
33mer
NA
NA
NA
NA
NA
NA
NA
NA
NA
NA
NA
NA
NA
NA
BW208 WT
-36
-36
-24
-3
-9
-39
-25
-78
-111
-74
-23
-61
-1
Delections
1 10 30 50 70 90 110 130
 I I I I I I I I
+83
+36
+47
+47
+51
-6
-11
-2
Insertions
b
PAM+sgAlpha-2
DQ2.5-glia-α3
33mer
1 10 20 90
I I I I
p31-43
NA
NA
NA
NA
THA53 WT
-18
-36
-36
-36
-19
-23
-10
Delections
c
PAM+sgAlpha-2
DQ2.5-glia-α3
1 10 20 90
I I I I
33mer
p31-43
NA
NA
NA
NA
NA
NA
DP WT
-1
-5
-12
-10
-15
-23
-36
Delections
1 10 30 50 70 90 110 130
 I I I I I I I I
+166
+166
Insertions
Supplementary Figure 2
