# Supplementary material for: Low‐gluten, nontransgenic wheat engineered with CRISPR/Cas9
Source: Plant Biotechnol J. 2017 Nov 24;16(4):902–10. doi: 10.1111/pbi.12837 (PMC5867031; doi:10.1111/pbi.12837)
Supplement: Supplementary file 8 — Figure S8 Gliadin and glutenin protein fractions analysed by A‐PAGE and SDS‐PAGE from T1 half‐seeds derived from T0 lines transformed with sgAlpha‐1 and sgAlpha‐2 constructs. [file PBI-16-902-s003.pptx]

## Slide 1
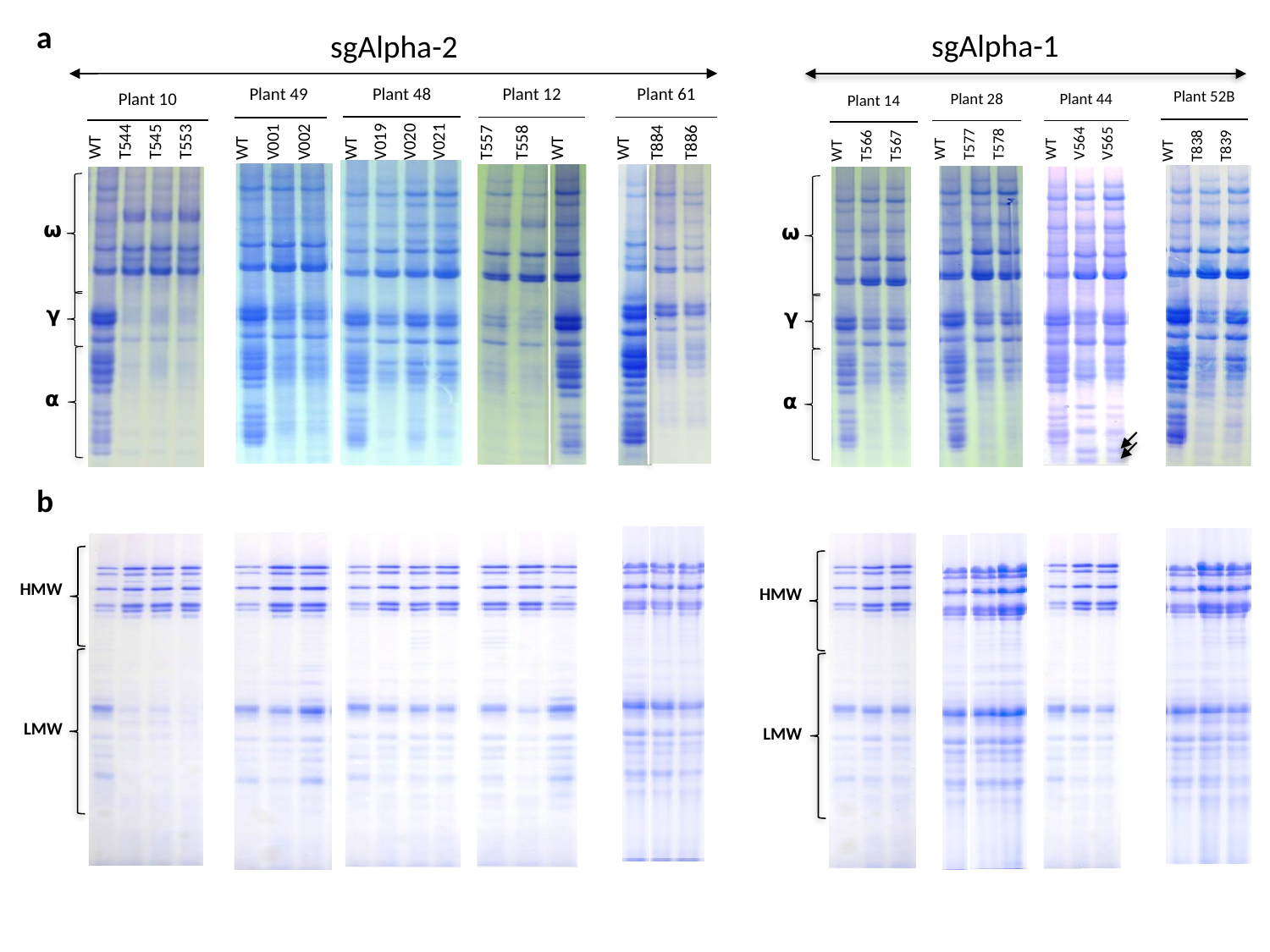

a
sgAlpha-1
sgAlpha-2
| Plant 48 | | | |
| --- | --- | --- | --- |
| WT | V019 | V020 | V021 |
| Plant 12 | | |
| --- | --- | --- |
| T557 | T558 | WT |
| Plant 61 | | |
| --- | --- | --- |
| WT | T884 | T886 |
| Plant 49 | | |
| --- | --- | --- |
| WT | V001 | V002 |
| Plant 52B | | |
| --- | --- | --- |
| WT | T838 | T839 |
| Plant 44 | | |
| --- | --- | --- |
| WT | V564 | V565 |
| Plant 28 | | |
| --- | --- | --- |
| WT | T577 | T578 |
| Plant 10 | | | |
| --- | --- | --- | --- |
| WT | T544 | T545 | T553 |
| Plant 14 | | |
| --- | --- | --- |
| WT | T566 | T567 |
𝞈
𝝲
𝝰
𝞈
𝝲
𝝰
b
HMW
LMW
HMW
LMW

## Slide 2
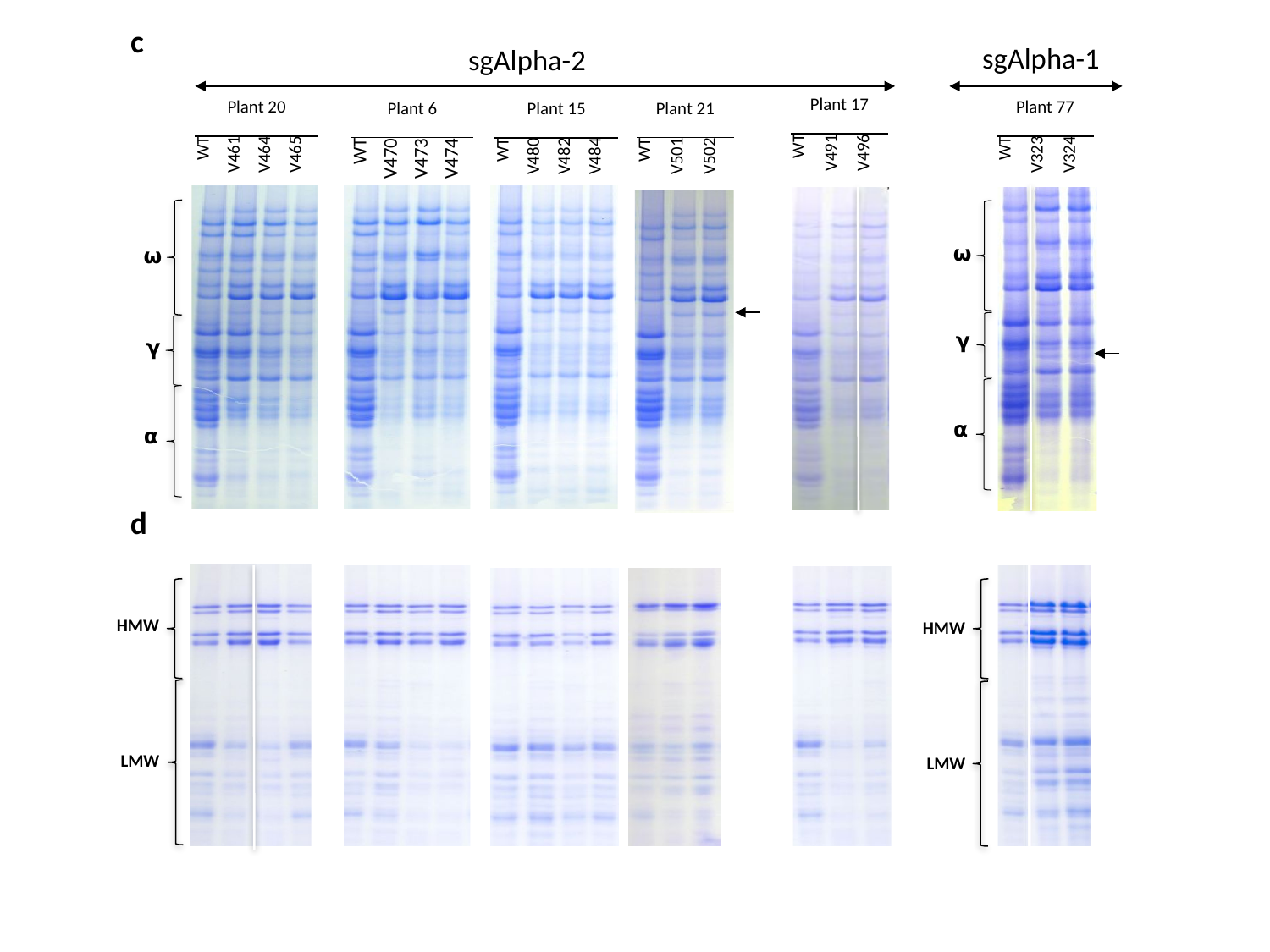

c
sgAlpha-1
sgAlpha-2
| Plant 17 | | |
| --- | --- | --- |
| WT | V491 | V496 |
| Plant 20 | | | |
| --- | --- | --- | --- |
| WT | V461 | V464 | V465 |
| Plant 77 | | |
| --- | --- | --- |
| WT | V323 | V324 |
| Plant 6 | | | |
| --- | --- | --- | --- |
| WT | V470 | V473 | V474 |
| Plant 21 | | |
| --- | --- | --- |
| WT | V501 | V502 |
| Plant 15 | | | |
| --- | --- | --- | --- |
| WT | V480 | V482 | V484 |
𝞈
𝝲
𝝰
𝞈
𝝲
𝝰
d
HMW
LMW
HMW
LMW

## Slide 3
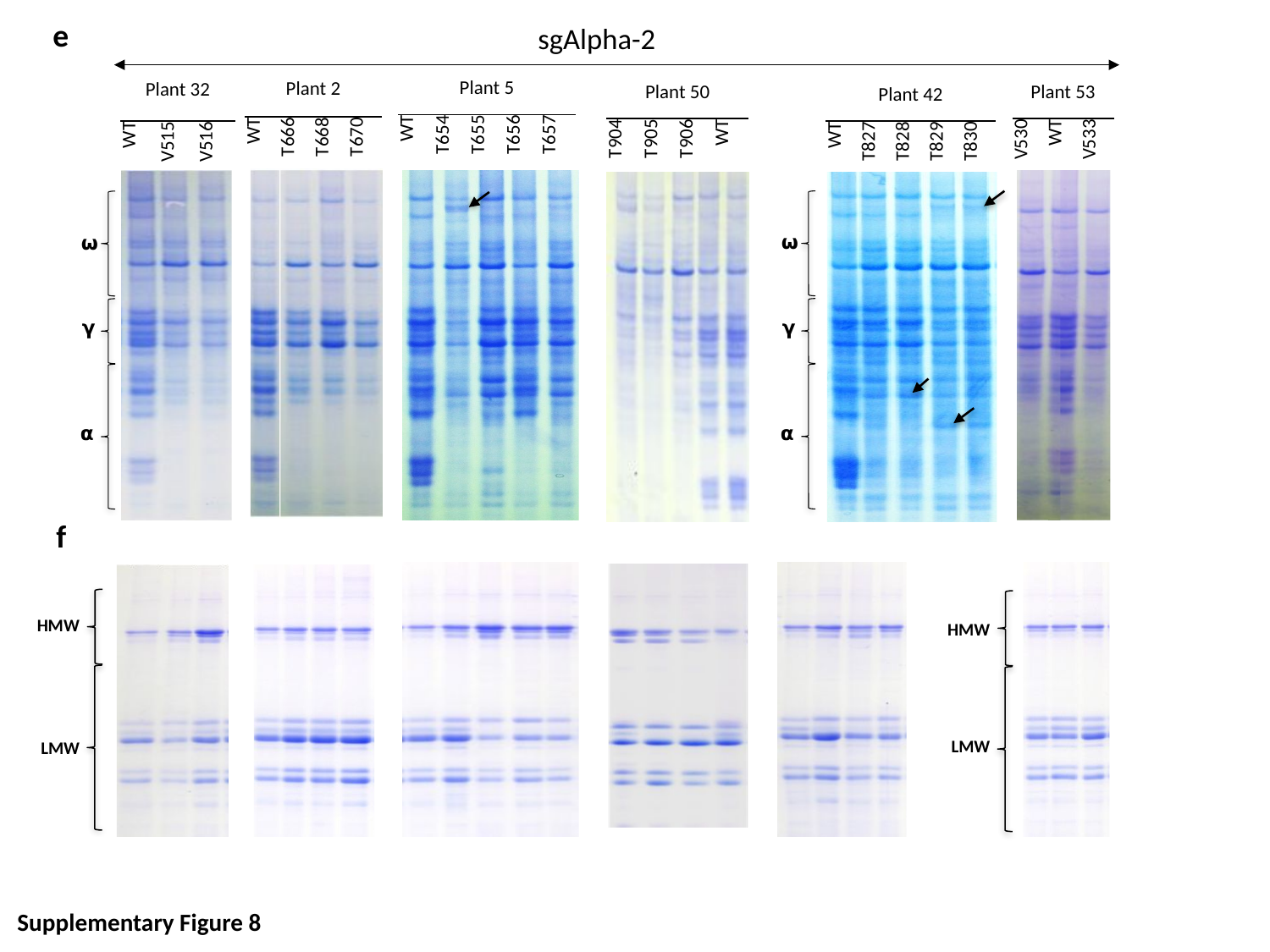

e
sgAlpha-2
| Plant 32 | | |
| --- | --- | --- |
| WT | V515 | V516 |
| Plant 2 | | | |
| --- | --- | --- | --- |
| WT | T666 | T668 | T670 |
| Plant 5 | | | | |
| --- | --- | --- | --- | --- |
| WT | T654 | T655 | T656 | T657 |
| Plant 50 | | | |
| --- | --- | --- | --- |
| T904 | T905 | T906 | WT |
| Plant 53 | | |
| --- | --- | --- |
| V530 | WT | V533 |
| Plant 42 | | | | |
| --- | --- | --- | --- | --- |
| WT | T827 | T828 | T829 | T830 |
𝞈
𝝲
𝝰
𝞈
𝝲
𝝰
f
HMW
LMW
HMW
LMW
Supplementary Figure 8
