# Supplementary material for: Low‐gluten, nontransgenic wheat engineered with CRISPR/Cas9
Source: Plant Biotechnol J. 2017 Nov 24;16(4):902–10. doi: 10.1111/pbi.12837 (PMC5867031; doi:10.1111/pbi.12837)
Supplement: Supplementary file 9 — Figure S9 Off‐target mutations detection in γ‐gliadin genes of BW208 wild type and two T1 mutant lines. [file PBI-16-902-s004.pptx]

## Slide 1
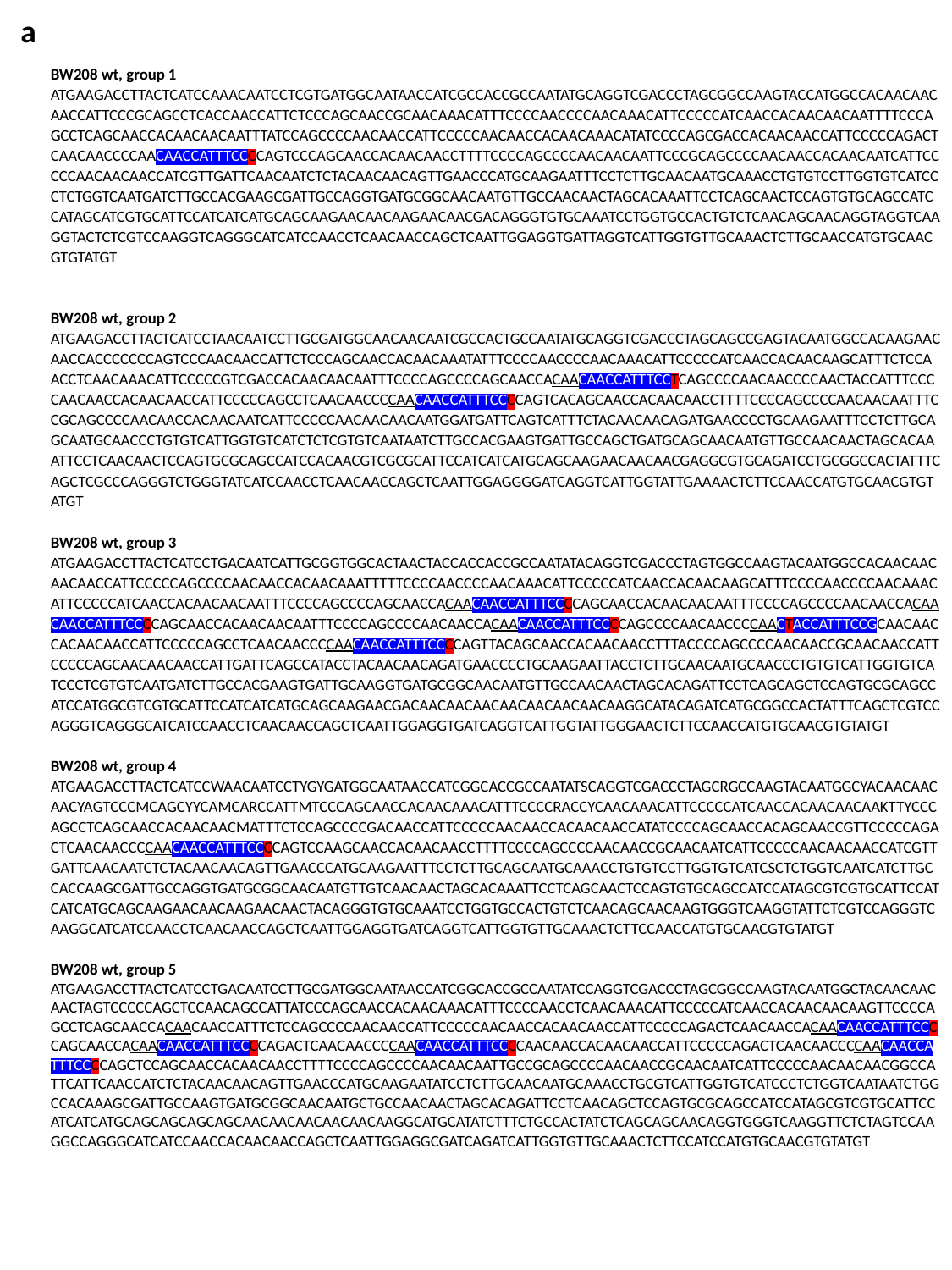

a
BW208 wt, group 1
ATGAAGACCTTACTCATCCAAACAATCCTCGTGATGGCAATAACCATCGCCACCGCCAATATGCAGGTCGACCCTAGCGGCCAAGTACCATGGCCACAACAACAACCATTCCCGCAGCCTCACCAACCATTCTCCCAGCAACCGCAACAAACATTTCCCCAACCCCAACAAACATTCCCCCATCAACCACAACAACAATTTTCCCAGCCTCAGCAACCACAACAACAATTTATCCAGCCCCAACAACCATTCCCCCAACAACCACAACAAACATATCCCCAGCGACCACAACAACCATTCCCCCAGACTCAACAACCCCAACAACCATTTCCCCAGTCCCAGCAACCACAACAACCTTTTCCCCAGCCCCAACAACAATTCCCGCAGCCCCAACAACCACAACAATCATTCCCCCAACAACAACCATCGTTGATTCAACAATCTCTACAACAACAGTTGAACCCATGCAAGAATTTCCTCTTGCAACAATGCAAACCTGTGTCCTTGGTGTCATCCCTCTGGTCAATGATCTTGCCACGAAGCGATTGCCAGGTGATGCGGCAACAATGTTGCCAACAACTAGCACAAATTCCTCAGCAACTCCAGTGTGCAGCCATCCATAGCATCGTGCATTCCATCATCATGCAGCAAGAACAACAAGAACAACGACAGGGTGTGCAAATCCTGGTGCCACTGTCTCAACAGCAACAGGTAGGTCAAGGTACTCTCGTCCAAGGTCAGGGCATCATCCAACCTCAACAACCAGCTCAATTGGAGGTGATTAGGTCATTGGTGTTGCAAACTCTTGCAACCATGTGCAACGTGTATGT
BW208 wt, group 2
ATGAAGACCTTACTCATCCTAACAATCCTTGCGATGGCAACAACAATCGCCACTGCCAATATGCAGGTCGACCCTAGCAGCCGAGTACAATGGCCACAAGAACAACCACCCCCCCAGTCCCAACAACCATTCTCCCAGCAACCACAACAAATATTTCCCCAACCCCAACAAACATTCCCCCATCAACCACAACAAGCATTTCTCCAACCTCAACAAACATTCCCCCGTCGACCACAACAACAATTTCCCCAGCCCCAGCAACCACAACAACCATTTCCTCAGCCCCAACAACCCCAACTACCATTTCCCCAACAACCACAACAACCATTCCCCCAGCCTCAACAACCCCAACAACCATTTCCCCAGTCACAGCAACCACAACAACCTTTTCCCCAGCCCCAACAACAATTTCCGCAGCCCCAACAACCACAACAATCATTCCCCCAACAACAACAATGGATGATTCAGTCATTTCTACAACAACAGATGAACCCCTGCAAGAATTTCCTCTTGCAGCAATGCAACCCTGTGTCATTGGTGTCATCTCTCGTGTCAATAATCTTGCCACGAAGTGATTGCCAGCTGATGCAGCAACAATGTTGCCAACAACTAGCACAAATTCCTCAACAACTCCAGTGCGCAGCCATCCACAACGTCGCGCATTCCATCATCATGCAGCAAGAACAACAACGAGGCGTGCAGATCCTGCGGCCACTATTTCAGCTCGCCCAGGGTCTGGGTATCATCCAACCTCAACAACCAGCTCAATTGGAGGGGATCAGGTCATTGGTATTGAAAACTCTTCCAACCATGTGCAACGTGTATGT
BW208 wt, group 3
ATGAAGACCTTACTCATCCTGACAATCATTGCGGTGGCACTAACTACCACCACCGCCAATATACAGGTCGACCCTAGTGGCCAAGTACAATGGCCACAACAACAACAACCATTCCCCCAGCCCCAACAACCACAACAAATTTTTCCCCAACCCCAACAAACATTCCCCCATCAACCACAACAAGCATTTCCCCAACCCCAACAAACATTCCCCCATCAACCACAACAACAATTTCCCCAGCCCCAGCAACCACAACAACCATTTCCCCAGCAACCACAACAACAATTTCCCCAGCCCCAACAACCACAACAACCATTTCCCCAGCAACCACAACAACAATTTCCCCAGCCCCAACAACCACAACAACCATTTCCCCAGCCCCAACAACCCCAACTACCATTTCCGCAACAACCACAACAACCATTCCCCCAGCCTCAACAACCCCAACAACCATTTCCCCAGTTACAGCAACCACAACAACCTTTACCCCAGCCCCAACAACCGCAACAACCATTCCCCCAGCAACAACAACCATTGATTCAGCCATACCTACAACAACAGATGAACCCCTGCAAGAATTACCTCTTGCAACAATGCAACCCTGTGTCATTGGTGTCATCCCTCGTGTCAATGATCTTGCCACGAAGTGATTGCAAGGTGATGCGGCAACAATGTTGCCAACAACTAGCACAGATTCCTCAGCAGCTCCAGTGCGCAGCCATCCATGGCGTCGTGCATTCCATCATCATGCAGCAAGAACGACAACAACAACAACAACAACAACAAGGCATACAGATCATGCGGCCACTATTTCAGCTCGTCCAGGGTCAGGGCATCATCCAACCTCAACAACCAGCTCAATTGGAGGTGATCAGGTCATTGGTATTGGGAACTCTTCCAACCATGTGCAACGTGTATGT
BW208 wt, group 4
ATGAAGACCTTACTCATCCWAACAATCCTYGYGATGGCAATAACCATCGGCACCGCCAATATSCAGGTCGACCCTAGCRGCCAAGTACAATGGCYACAACAACAACYAGTCCCMCAGCYYCAMCARCCATTMTCCCAGCAACCACAACAAACATTTCCCCRACCYCAACAAACATTCCCCCATCAACCACAACAACAAKTTYCCCAGCCTCAGCAACCACAACAACMATTTCTCCAGCCCCGACAACCATTCCCCCAACAACCACAACAACCATATCCCCAGCAACCACAGCAACCGTTCCCCCAGACTCAACAACCCCAACAACCATTTCCCCAGTCCAAGCAACCACAACAACCTTTTCCCCAGCCCCAACAACCGCAACAATCATTCCCCCAACAACAACCATCGTTGATTCAACAATCTCTACAACAACAGTTGAACCCATGCAAGAATTTCCTCTTGCAGCAATGCAAACCTGTGTCCTTGGTGTCATCSCTCTGGTCAATCATCTTGCCACCAAGCGATTGCCAGGTGATGCGGCAACAATGTTGTCAACAACTAGCACAAATTCCTCAGCAACTCCAGTGTGCAGCCATCCATAGCGTCGTGCATTCCATCATCATGCAGCAAGAACAACAAGAACAACTACAGGGTGTGCAAATCCTGGTGCCACTGTCTCAACAGCAACAAGTGGGTCAAGGTATTCTCGTCCAGGGTCAAGGCATCATCCAACCTCAACAACCAGCTCAATTGGAGGTGATCAGGTCATTGGTGTTGCAAACTCTTCCAACCATGTGCAACGTGTATGT
BW208 wt, group 5
ATGAAGACCTTACTCATCCTGACAATCCTTGCGATGGCAATAACCATCGGCACCGCCAATATCCAGGTCGACCCTAGCGGCCAAGTACAATGGCTACAACAACAACTAGTCCCCCAGCTCCAACAGCCATTATCCCAGCAACCACAACAAACATTTCCCCAACCTCAACAAACATTCCCCCATCAACCACAACAACAAGTTCCCCAGCCTCAGCAACCACAACAACCATTTCTCCAGCCCCAACAACCATTCCCCCAACAACCACAACAACCATTCCCCCAGACTCAACAACCACAACAACCATTTCCCCAGCAACCACAACAACCATTTCCCCAGACTCAACAACCCCAACAACCATTTCCCCAACAACCACAACAACCATTCCCCCAGACTCAACAACCCCAACAACCATTTCCCCAGCTCCAGCAACCACAACAACCTTTTCCCCAGCCCCAACAACAATTGCCGCAGCCCCAACAACCGCAACAATCATTCCCCCAACAACAACGGCCATTCATTCAACCATCTCTACAACAACAGTTGAACCCATGCAAGAATATCCTCTTGCAACAATGCAAACCTGCGTCATTGGTGTCATCCCTCTGGTCAATAATCTGGCCACAAAGCGATTGCCAAGTGATGCGGCAACAATGCTGCCAACAACTAGCACAGATTCCTCAACAGCTCCAGTGCGCAGCCATCCATAGCGTCGTGCATTCCATCATCATGCAGCAGCAGCAGCAACAACAACAACAACAAGGCATGCATATCTTTCTGCCACTATCTCAGCAGCAACAGGTGGGTCAAGGTTCTCTAGTCCAAGGCCAGGGCATCATCCAACCACAACAACCAGCTCAATTGGAGGCGATCAGATCATTGGTGTTGCAAACTCTTCCATCCATGTGCAACGTGTATGT

## Slide 2
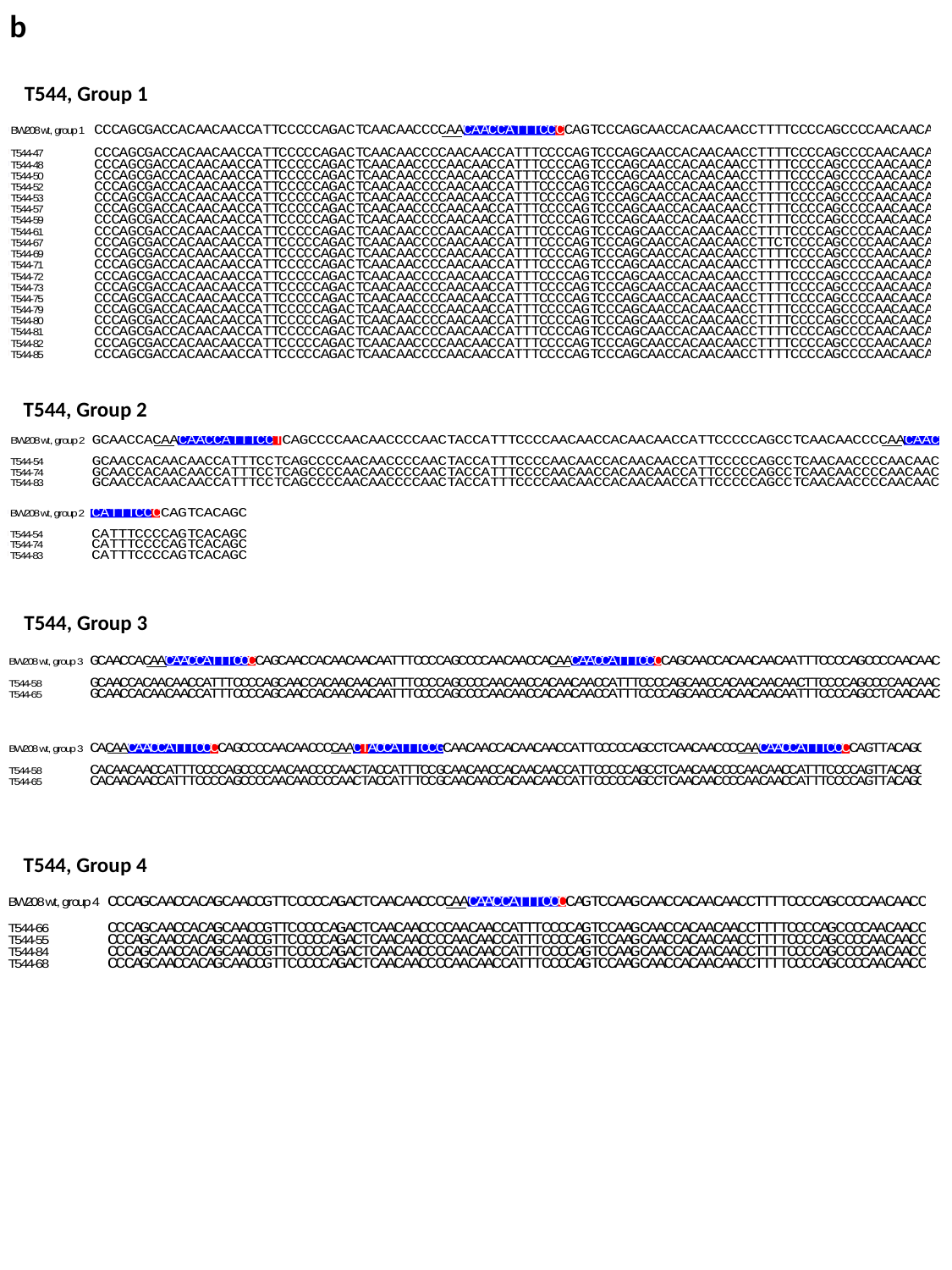

b
T544, Group 1
T544, Group 2
T544, Group 3
T544, Group 4

## Slide 3
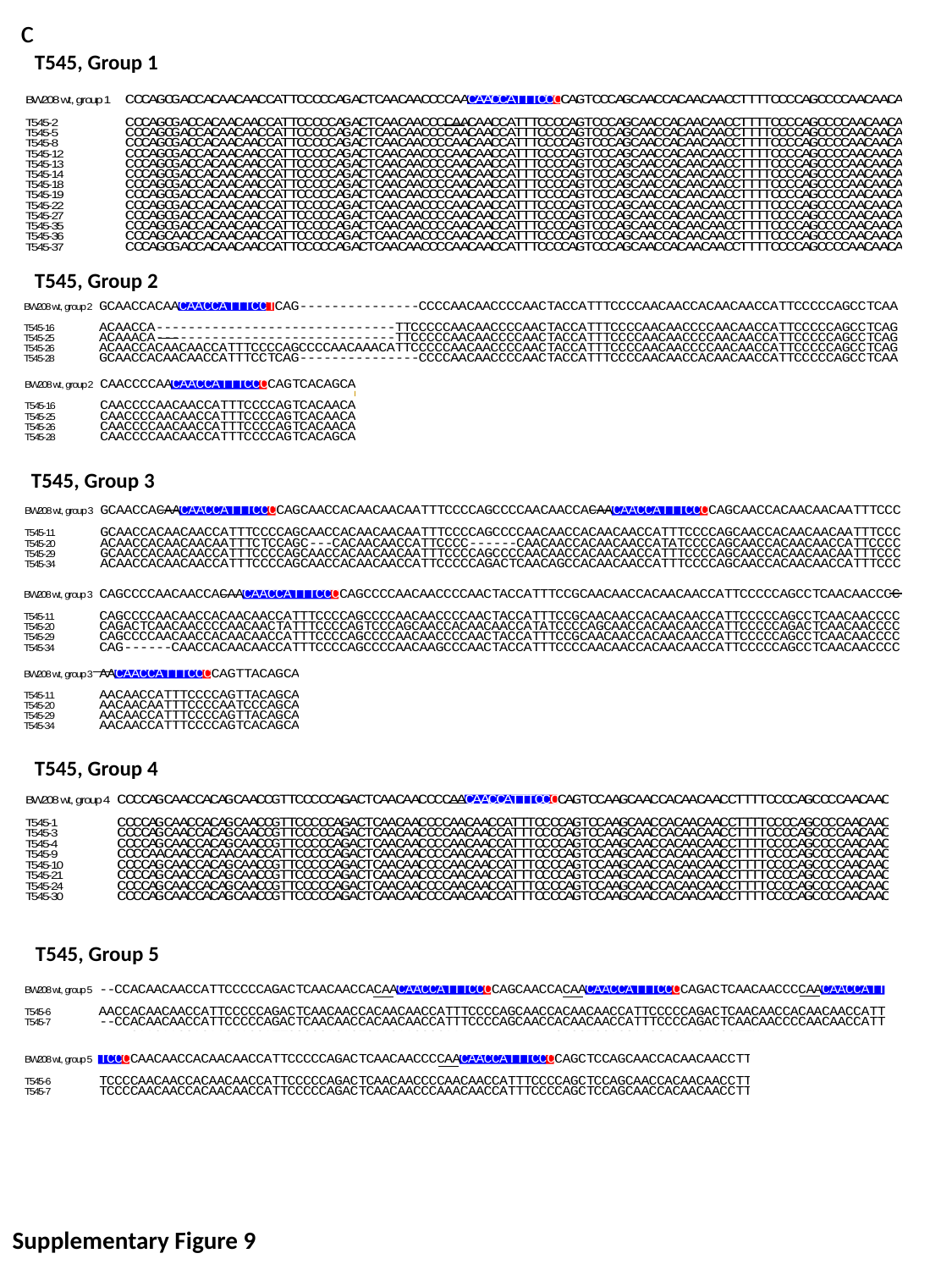

c
T545, Group 1
T545, Group 2
T545, Group 3
T545, Group 4
T545, Group 5
Supplementary Figure 9
