# Supplementary material for: Low‐gluten, nontransgenic wheat engineered with CRISPR/Cas9
Source: Plant Biotechnol J. 2017 Nov 24;16(4):902–10. doi: 10.1111/pbi.12837 (PMC5867031; doi:10.1111/pbi.12837)
Supplement: Supplementary file 12 — Figure S12 Off‐target mutations detection in BW208 mutant lines. [file PBI-16-902-s007.pptx]

## Slide 1
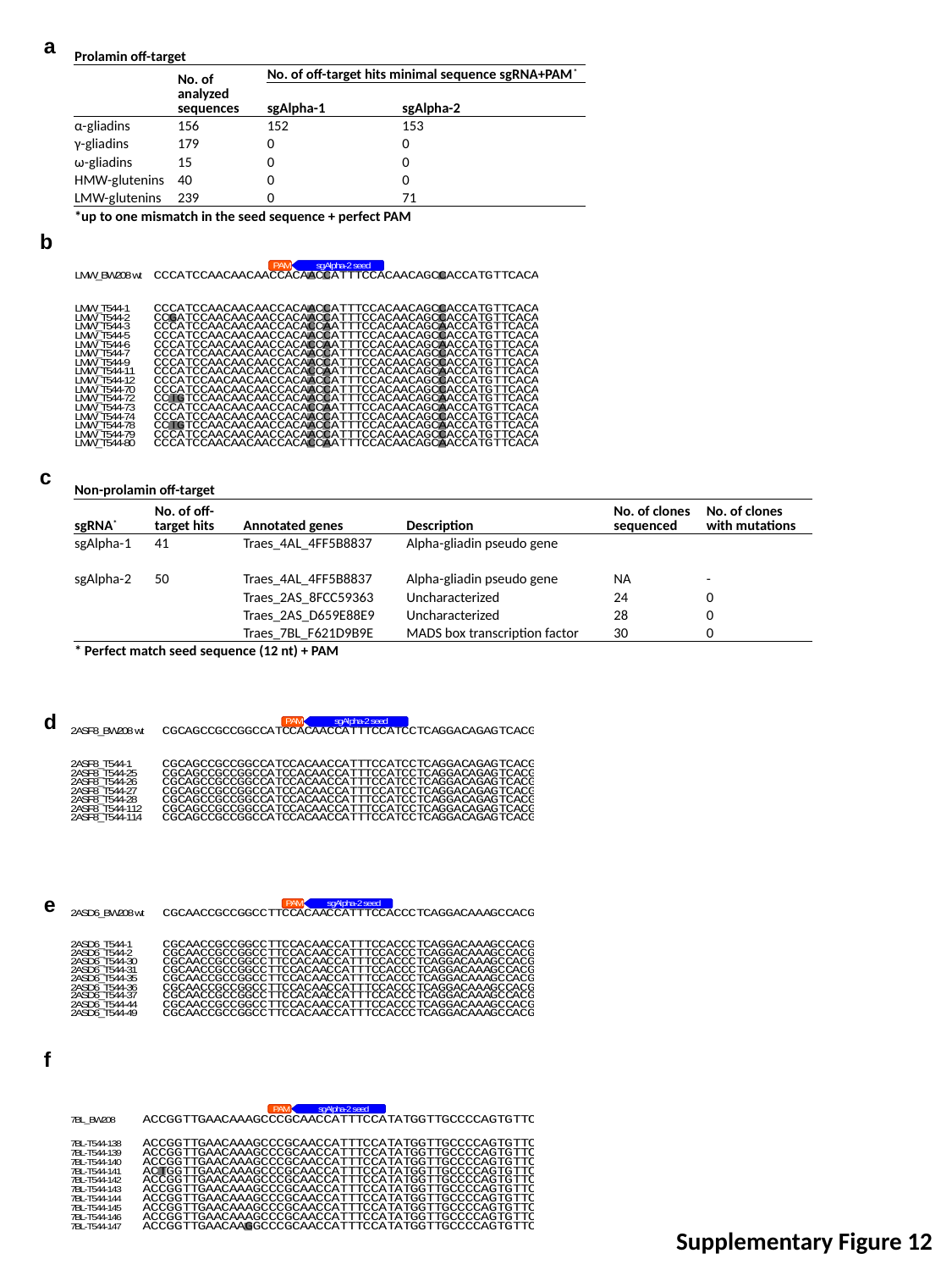

a
| Prolamin off-target | | | |
| --- | --- | --- | --- |
| | No. of analyzed sequences | No. of off-target hits minimal sequence sgRNA+PAM\* | |
| | | sgAlpha-1 | sgAlpha-2 |
| α-gliadins | 156 | 152 | 153 |
| γ-gliadins | 179 | 0 | 0 |
| ω-gliadins | 15 | 0 | 0 |
| HMW-glutenins | 40 | 0 | 0 |
| LMW-glutenins | 239 | 0 | 71 |
| \*up to one mismatch in the seed sequence + perfect PAM | | | |
b
c
| Non-prolamin off-target | | | | | | |
| --- | --- | --- | --- | --- | --- | --- |
| sgRNA\* | No. of off-target hits | Annotated genes | Description | | No. of clones sequenced | No. of clones with mutations |
| sgAlpha-1 | 41 | Traes\_4AL\_4FF5B8837 | Alpha-gliadin pseudo gene | | | |
| | | | | | | |
| sgAlpha-2 | 50 | Traes\_4AL\_4FF5B8837 | Alpha-gliadin pseudo gene | | NA | - |
| | | Traes\_2AS\_8FCC59363 | Uncharacterized | | 24 | 0 |
| | | Traes\_2AS\_D659E88E9 | Uncharacterized | | 28 | 0 |
| | | Traes\_7BL\_F621D9B9E | MADS box transcription factor | | 30 | 0 |
| \* Perfect match seed sequence (12 nt) + PAM | | | | | | |
d
e
f
Supplementary Figure 12
