# Supplementary material for: Low‐gluten, nontransgenic wheat engineered with CRISPR/Cas9
Source: Plant Biotechnol J. 2017 Nov 24;16(4):902–10. doi: 10.1111/pbi.12837 (PMC5867031; doi:10.1111/pbi.12837)
Supplement: Supplementary file 13 — Figure S13 Multisite Gateway cloning of pANIC6E‐CR‐Alpha1 vector. [file PBI-16-902-s008.pptx]

## Slide 1
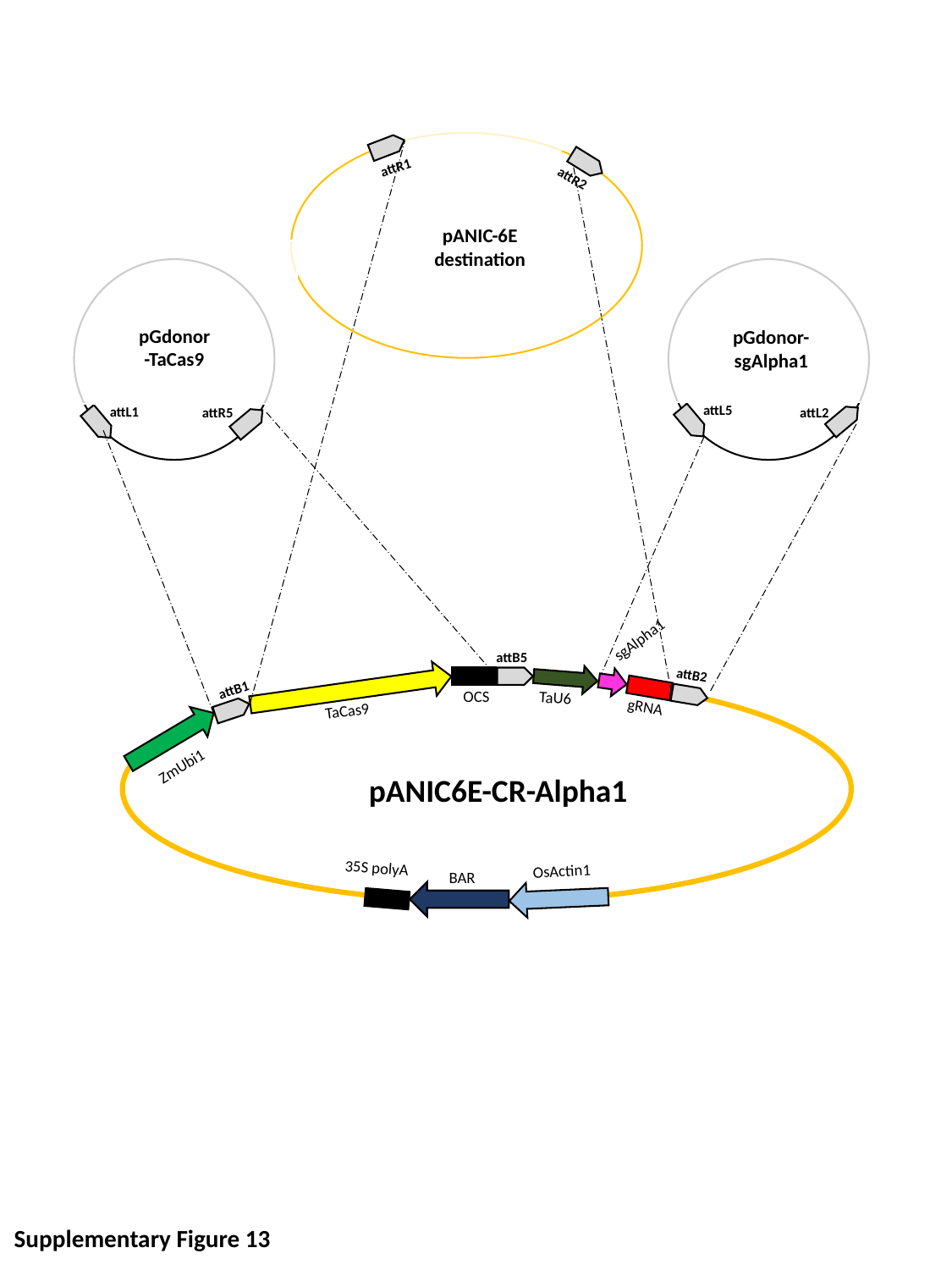

attR1
attR2
pANIC-6E
destination
pGdonor-TaCas9
pGdonor-sgAlpha1
attL5
attL1
attR5
attL2
sgAlpha1
attB5
attB2
attB1
OCS
TaU6
gRNA
TaCas9
ZmUbi1
pANIC6E-CR-Alpha1
35S polyA
OsActin1
BAR
Supplementary Figure 13
