# Supplementary material for: Low‐gluten, nontransgenic wheat engineered with CRISPR/Cas9
Source: Plant Biotechnol J. 2017 Nov 24;16(4):902–10. doi: 10.1111/pbi.12837 (PMC5867031; doi:10.1111/pbi.12837)
Supplement: Supplementary file 14 — Figure S14 Analysis by PCR and Illumina high‐throughput sequencing for the presence of the plasmid DNA; bar and Cas9 genes, PVS1 stability (sta) region, Octopine synthase polyA signal, and Panicum virgatum ubiquitin 1 promoter; and insertions in sgAlpha‐2 derived lines. [file PBI-16-902-s009.pptx]

## Slide 1
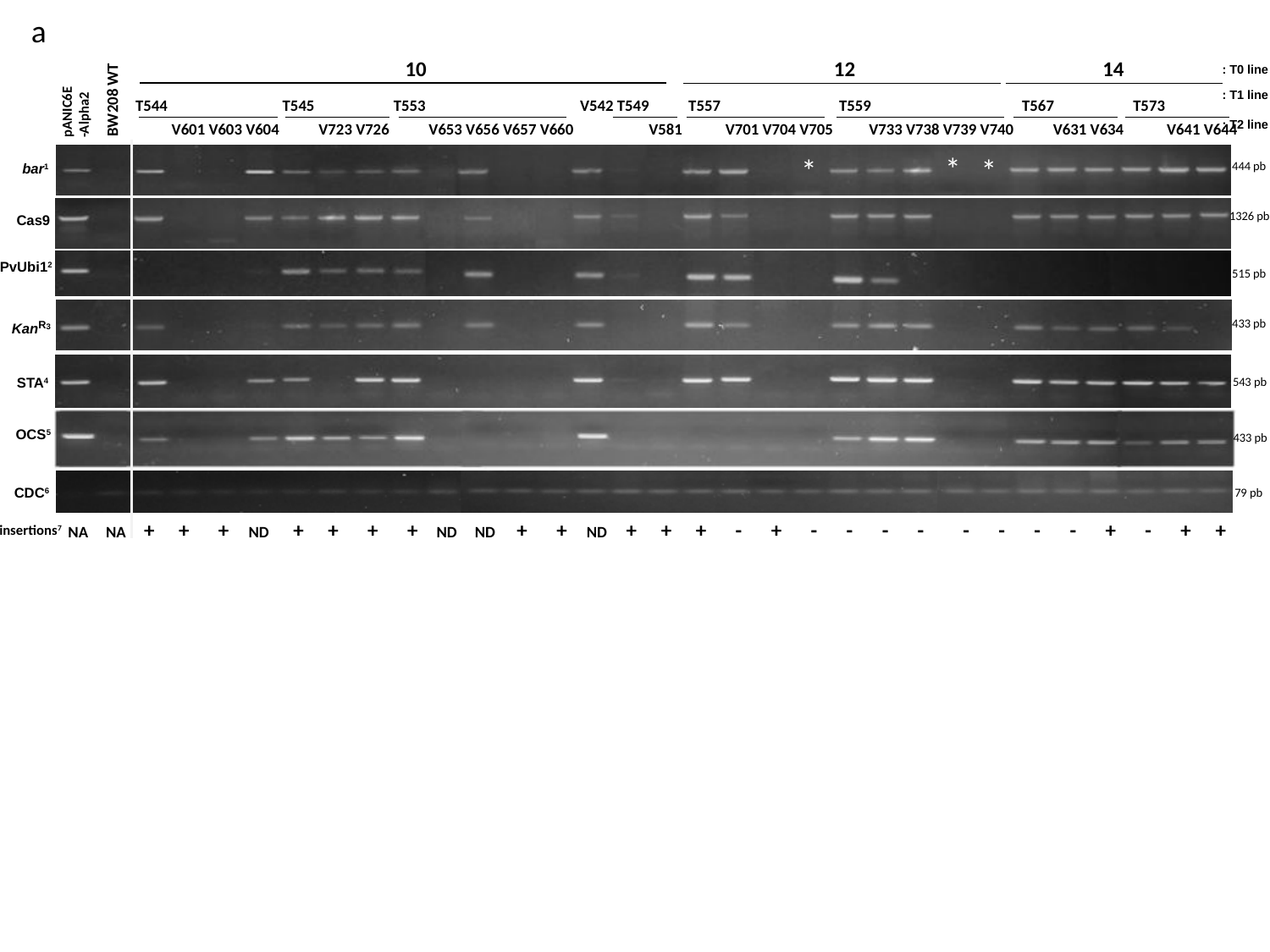

a
| : T0 line |
| --- |
| : T1 line |
| : T2 line |
10 12 14
BW208 WT
pANIC6E
-Alpha2
 T544 T545 T553 V542 T549 T557 T559 T567 T573
 V601 V603 V604 V723 V726 V653 V656 V657 V660 V581 V701 V704 V705 V733 V738 V739 V740 V631 V634 V641 V644
*
*
*
444 pb
bar1
insertions7
1326 pb
Cas9
PvUbi12
515 pb
KanR3
433 pb
STA4
543 pb
OCS5
433 pb
CDC6
79 pb
 NA NA + + + ND + + + + ND ND + + ND + + + - + - - - - - - - - + - + +

## Slide 2
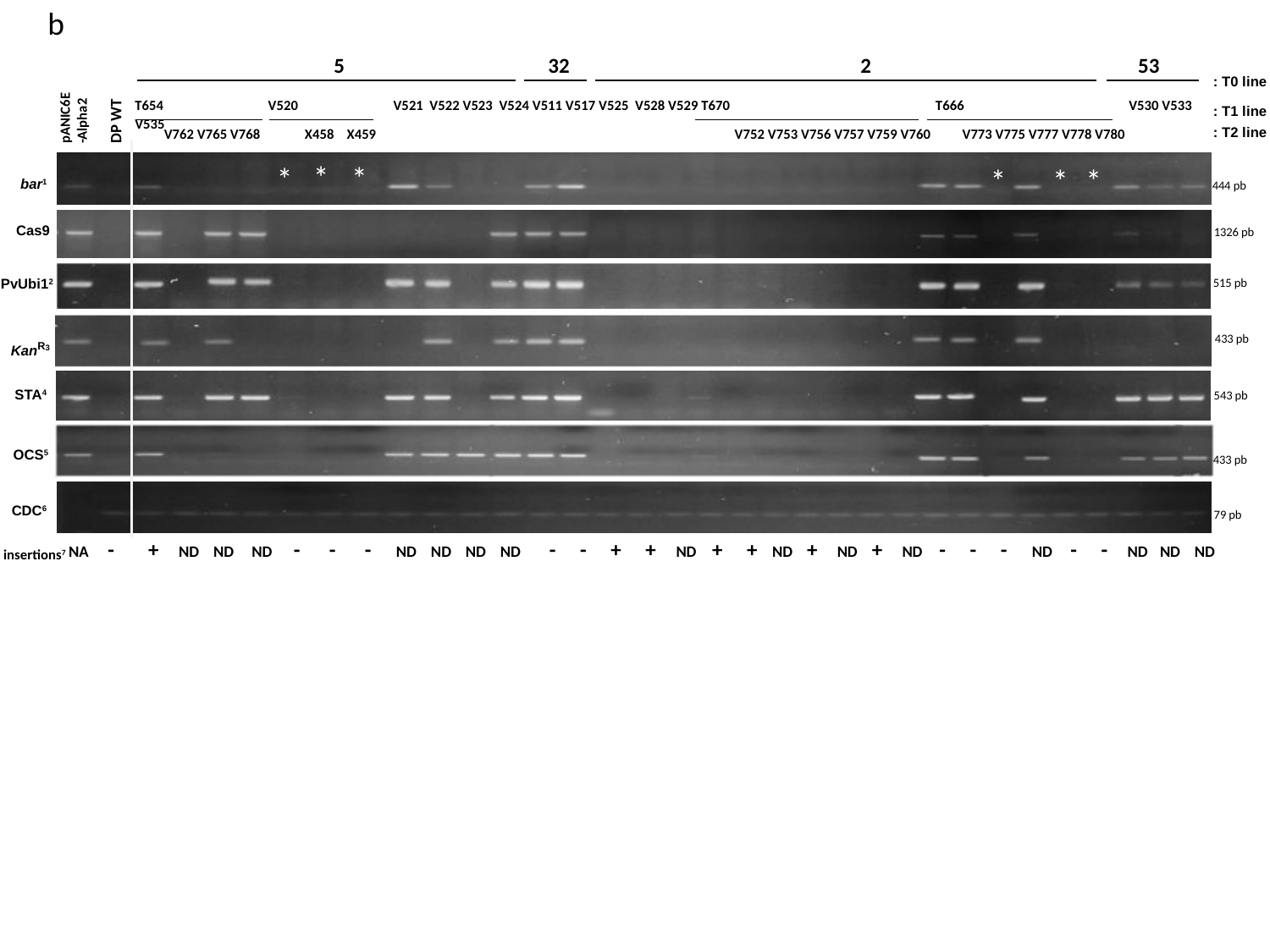

b
| : T0 line |
| --- |
| : T1 line |
| : T2 line |
5 32 2 53
pANIC6E
-Alpha2
DP WT
T654 V520 V521 V522 V523 V524 V511 V517 V525 V528 V529 T670 T666 V530 V533 V535
 V762 V765 V768 X458 X459 V752 V753 V756 V757 V759 V760 V773 V775 V777 V778 V780
*
*
*
*
*
*
bar1
insertions7
444 pb
Cas9
1326 pb
PvUbi12
515 pb
433 pb
KanR3
STA4
543 pb
OCS5
433 pb
CDC6
79 pb
NA - + ND ND ND - - - ND ND ND ND - - + + ND + + ND + ND + ND - - - ND - - ND ND ND

## Slide 3
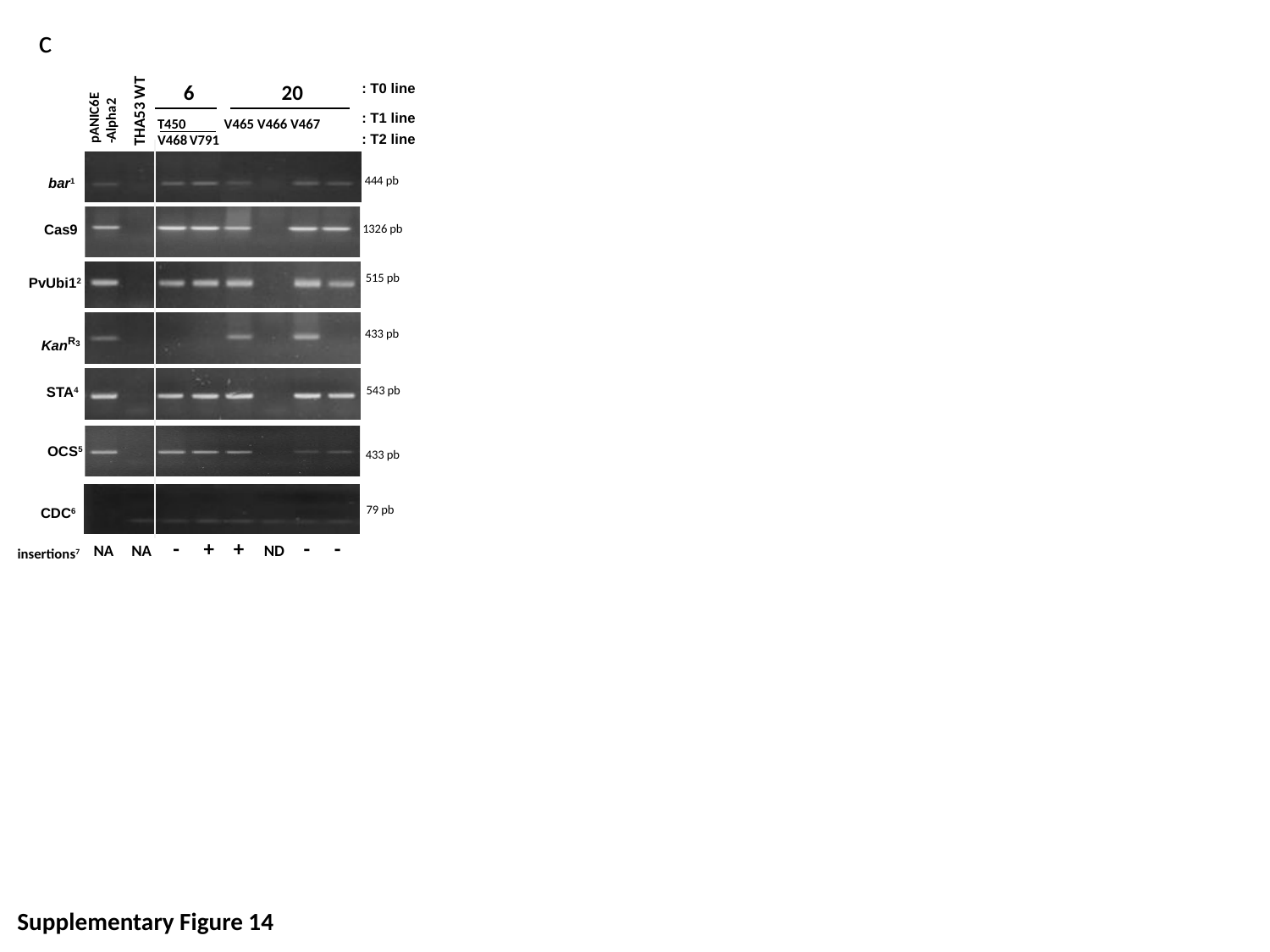

c
| : T0 line |
| --- |
| : T1 line |
| : T2 line |
6 20
pANIC6E
-Alpha2
THA53 WT
T450 V465 V466 V467 V468
 V791
444 pb
bar1
insertions7
1326 pb
Cas9
515 pb
PvUbi12
433 pb
KanR3
543 pb
STA4
OCS5
433 pb
79 pb
CDC6
NA NA - + + ND - -
Supplementary Figure 14
