# Supplementary material for: Low‐gluten, nontransgenic wheat engineered with CRISPR/Cas9
Source: Plant Biotechnol J. 2017 Nov 24;16(4):902–10. doi: 10.1111/pbi.12837 (PMC5867031; doi:10.1111/pbi.12837)
Supplement: Supplementary file 15 — Figure S15 Protein analysis of non‐transgenic transgenic (transgene‐free and insertion‐free) lines determined by RP‐HPLC and A‐PAGE gels. [file PBI-16-902-s010.pptx]

## Slide 1
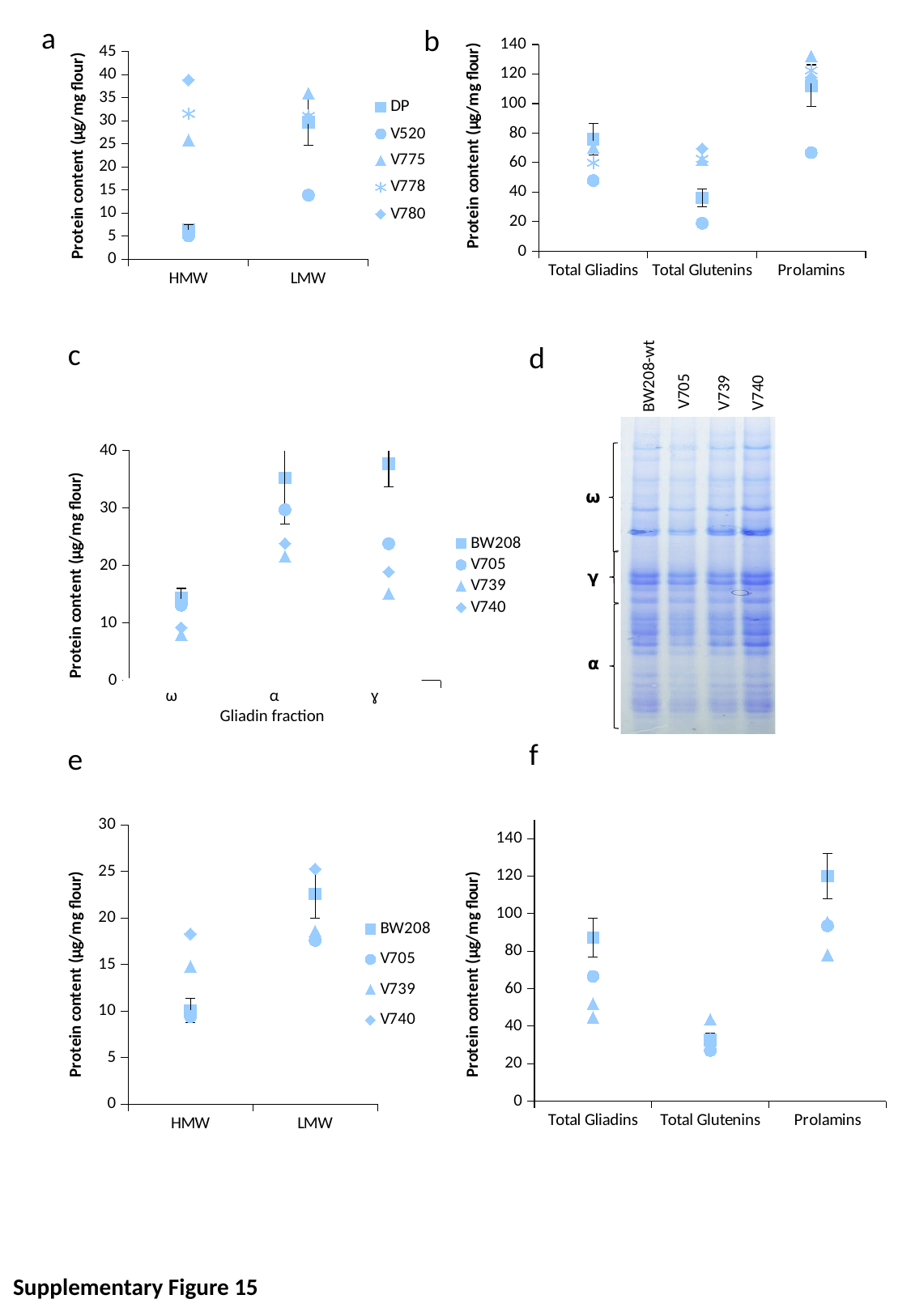

a
b
### Chart
| Category | DP | V520 | V775 | V778 | V780 |
|---|---|---|---|---|---|
| Total Gliadins | 75.9871114859876 | 47.84160595202064 | 70.22945969450905 | 59.92685593463473 | 48.1129003698521 |
| Total Glutenins | 36.04922373908769 | 18.92947173449754 | 61.7946082824559 | 62.6271693720532 | 69.55227604753851 |
| Prolamins | 112.0363352250752 | 66.77107768651817 | 132.024067976965 | 122.5540253066879 | 117.6651764173907 |
### Chart
| Category | DP | V520 | V775 | V778 | V780 |
|---|---|---|---|---|---|
| HMW | 6.391512553895607 | 5.031883398975231 | 25.83145744889262 | 31.54670744630078 | 38.85838410214409 |
| LMW | 29.65771118519207 | 13.89758833552231 | 35.96315083356327 | 31.08046192575242 | 30.69389194539447 |BW208-wt
V705
V740
V739
𝞈
𝝲
𝝰
c
d
### Chart
| Category | BW208 | V705 | V739 | V740 |
|---|---|---|---|---|
| Omega | 14.34351494436574 | 13.070739564033 | 7.92017479507443 | 9.19517504512034 |
| Alpha | 35.22532556239822 | 29.68225506387431 | 21.5707825106111 | 23.82769299415809 |
| Gamma | 37.6778633897729 | 23.7728737559931 | 15.10350663591011 | 18.87102957115358 | ω α ɣ
Gliadin fraction
f
e
### Chart
| Category | BW208 | V705 | V739 | V740 |
|---|---|---|---|---|
| HMW | 10.0531051050521 | 9.392625129848406 | 14.79136416657307 | 18.28338821166437 |
| LMW | 22.59464407347845 | 17.58519695230508 | 18.57011220102317 | 25.27925643696022 |
### Chart
| Category | BW208 | V705 | V739 | V740 |
|---|---|---|---|---|
| Total Gliadins | 87.24670389653683 | 66.52586838390036 | 44.59446394159565 | 51.893897610432 |
| Total Glutenins | 32.64774917853055 | 26.9778220821535 | 33.36147636759624 | 43.5626446486246 |
| Prolamins | 119.8944530750674 | 93.50369046605391 | 77.95594030919187 | 95.45654225905658 |Supplementary Figure 15
